# Activity evaluation of multifunctional H_2_S donors for anti-inflammatory, cardioprotective, and hepatoprotective applications

**DOI:** 10.3389/fchem.2025.1643663

**Published:** 2025-09-05

**Authors:** Donghe Wang, Yujie Meng, Yihong Liu

**Affiliations:** The Second Hospital of Qinhuangdao, Qinhuangdao, China

**Keywords:** hydrogen sulfide, cardioprotective, hepatoprotective, anti-inflammatory, phenylthiophosphonic dichloride

## Abstract

**Introduction:**

As an important gas signaling molecule, hydrogen sulfide (H_2_S) exhibits therapeutic potential in inflammatory and oxidative stress-related diseases. This study developed and evaluated novel H_2_S donor derivatives based on the phenylphosphonothioic dichloride scaffold.

**Methods:**

Derivatives were synthesized based on the phenylphosphonothioic dichloride scaffold. Compound **3b‐1** was selected for its high H_2_S release capacity and favorable safety profile. Its anti-inflammatory activity was evaluated by measuring inhibition of TNF‐α, TNF‐β, and nitrite. Hepatoprotective effects were assessed in an H_2_O_2_‐induced injury model using oxidative stress markers (MDA, SOD, GSH) and HSC activation. Cardioprotective effects were examined in an LPS-induced model by analyzing mitochondrial membrane potential, cardiac markers (LDH, CK‐MB), and oxidative balance.

**Results:**

Compound **3b-1** showed the highest H2S release capacity and inhibited TNF‐α (86%), TNF‐β (82%), and nitrite (67%). In the hepatocyte model, it reduced MDA (79%), enhanced SOD (49%) and GSH (76%), and suppressed HSC activation (55%). In the myocardial model, **3b‐1** attenuated mitochondrial membrane potential dissipation, decreased LDH (34%) and CK-MB (24%), and restored GSH activity (73%) while reducing MDA (48%).

**Discussion:**

The phosphorus-sulfur scaffold‐based H_2_S donor **3b‐1** demonstrates synergistic anti-inflammatory, antioxidant, and organ-protective effects, highlighting its promise as a drug candidate for treating inflammation- and oxidative stress-related disorders.

## 1 Introduction

**FIGURE 1:**
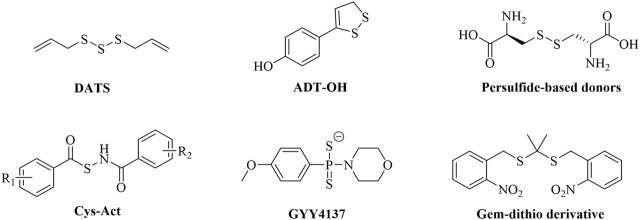
Representative H_2_S donor compounds.

Hydrogen sulfide (H_2_S) has been identified as the third endogenous gaseous signaling molecule, following nitric oxide (NO) and carbon monoxide (CO). In recent years, it has been demonstrated to be widely involved in physiological and pathological processes such as cardiovascular homeostasis regulation, inflammation suppression, and hepatocyte metabolic regulation (Jin et al., 2024; Yang et al., 2020). Regarding the cardiovascular system, H_2_S inhibits vascular smooth muscle cell proliferation by activating ATP-sensitive potassium channels, modulates myocardial ion channel function, and attenuates ischemia-reperfusion injury (Kang et al., 2016). Furthermore, H_2_S has been shown to reduce myocardial fibrosis and collagen deposition within atherosclerotic plaques by inhibiting the Transforming Growth Factor Beta 1 (TGF-β1) signaling pathway, thereby delaying the process of vascular remodeling (Huang et al., 2023). In the field of inflammation regulation, low concentrations of H_2_S suppress the expression of pro-inflammatory factors such as Tumor Necrosis Factor Alpha (TNF-α) and Tumor Necrosis Factor Beta (TNF-β) by inhibiting the Nuclear Factor kappa-B (NF-κB) pathway (Lu and Wen, 2025). In the area of myocardial protection, the core mechanism of H_2_S lies in its dual capability to counteract apoptosis and promote tissue repair (Salloum, 2015). Despite this, current research on H_2_S donors still faces three major bottlenecks: poor chemical stability (e.g., the commonly used donor NaHS has an extremely short half-life), lack of tissue targeting, and uncontrollable release kinetics. An ideal H_2_S donor should release H_2_S only upon specific activation and with controlled, slow kinetics. To address this need, chemists have developed multiple novel types of H_2_S donors over the past decade that can be activated in response to different triggers, such as hydrolysis, biological thiols, light, pH, and enzymes (Figure 1) (Zhao et al., 2017). Major types include: natural donors (e.g., diallyl trisulfide (DATS) from garlic extracts) that release H_2_S in the presence of biological thiols (e.g., glutathione, GSH) and glucose (Chuah et al., 2007); hydrolysis-activated donors (e.g., ADT-OH) that primarily release H_2_S via hydrolysis (Wallace et al., 2007); persulfide donors that rapidly release H_2_S predominantly through exchange reactions with endogenous thiols (e.g., GSH) (Szczesny et al., 2014); thiol-triggered donors (e.g., Cys-Act) where H_2_S release occurs following nucleophilic addition by a thiol (Zhao et al., 2011); slow-release donors (e.g., GYY4137) that release H_2_S upon hydrolysis with controlled kinetics (Park et al., 2013); and photo-induced donors (e.g., gem-dithiol derivatives) where UV light exposure cleaves a photolabile protecting group, followed by hydrolysis to release H_2_S (Devarie-Baez et al., 2013). However, these donors also have limitations, such as uncontrolled release kinetics, limited trigger specificity, and significant toxicity, among others. Consequently, developing a new generation of disease microenvironment-responsive, controlled-release, and non-toxic H_2_S donors has become imperative to overcome current therapeutic limitations.

**SCHEME 1:**
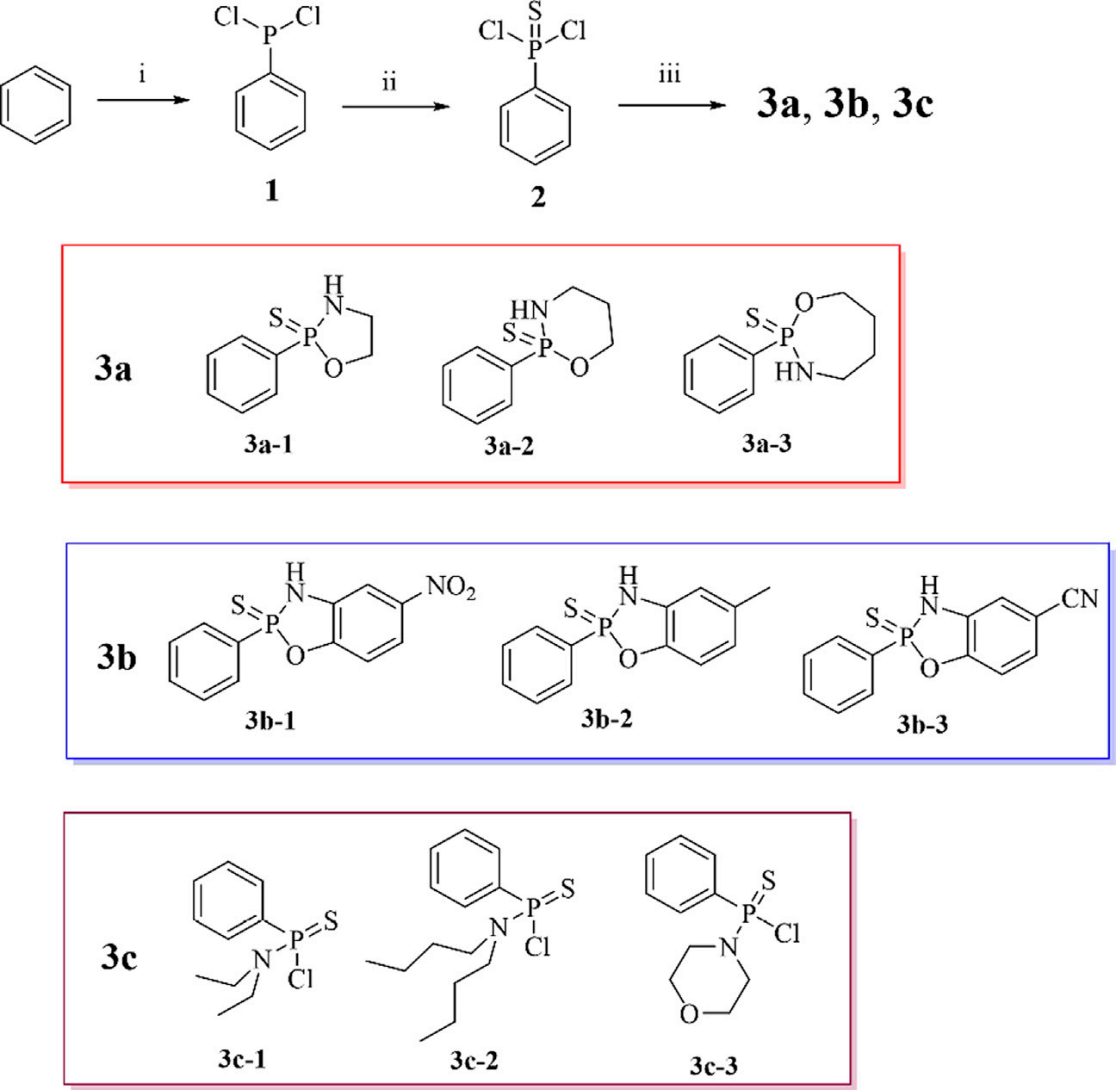
Synthesis of H_2_S donor. Conditions and reagents: (i) AlCl3, PCl3, 75%; (ii) S, Benzene, 40%; (iii) Triethylamine, Monoethanolamine, 3-Aminopropanol, 4-Amino-1-butanol, 2-Amino-4-nitrophenol, 2-Amino-p-cresol, 2-Amino-4-cyanophenol, Diethylamine, Ethylbutylamine, Morpholine, 30%–45%.

**FIGURE 2:**
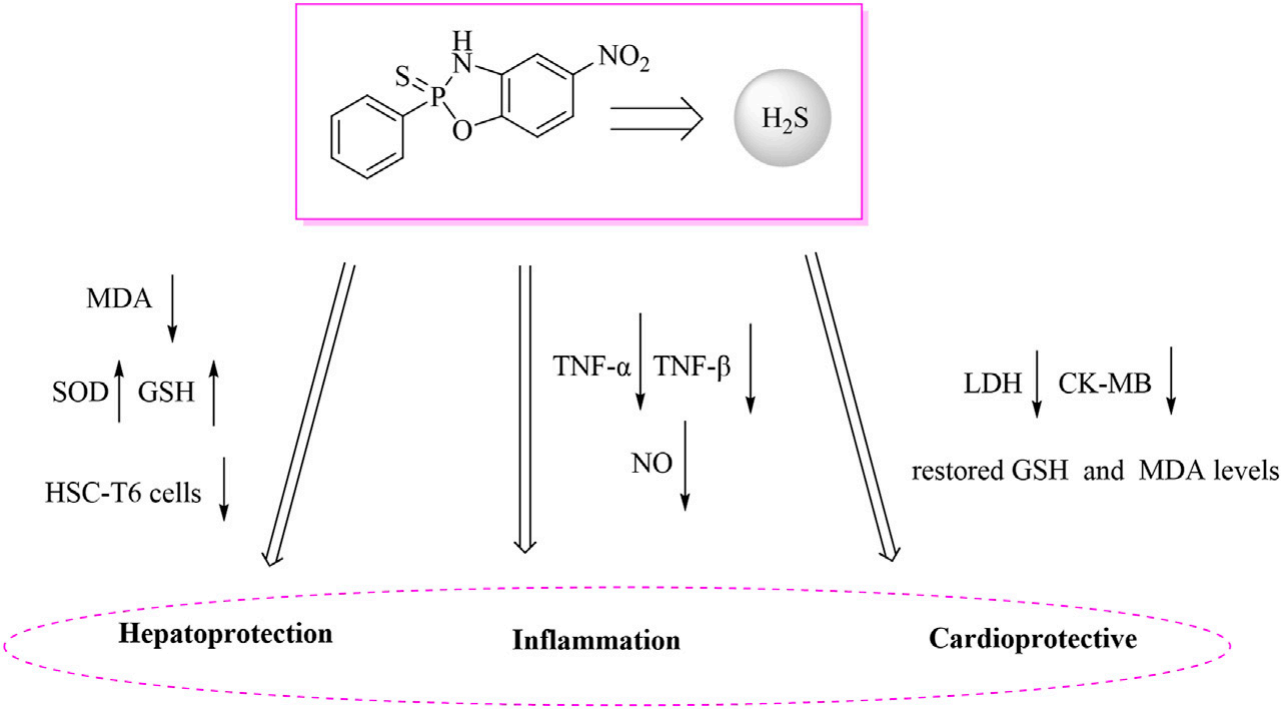
Schematic diagram of H_2_S partial immunomodulatory mechanism.

The liver, as the core organ for metabolism and detoxification, has its functional integrity vulnerable to damage from oxidative stress and fibrosis. H_2_S exhibits multi-layered physiological regulatory mechanisms in liver protection (Lee and Im, 2022). As illustrated in Figure 2, at the level of antioxidant defense, H_2_S effectively neutralizes reactive oxygen species (ROS) and lipid peroxidation products such as malondialdehyde (MDA) by enhancing glutathione (GSH) biosynthesis and boosting superoxide dismutase (SOD) activity. Studies demonstrate that exogenous H_2_S donors can significantly reduce MDA levels while elevating SOD and GSH in carbon tetrachloride-induced liver fibrosis models, thereby maintaining redox homeostasis (Cirino et al., 2023). Septic cardiomyopathy, a fatal complication in critically ill patients with severe infections, is characterized by mitochondrial dysfunction and a systemic inflammatory storm, and currently lacks specific therapeutic interventions (Zhou et al., 2022). Endotoxin lipopolysaccharide (LPS) activates Toll-like receptor 4 (TLR4), triggering NF-κB and NOD-like receptor pyrin domain-containing protein 3 (NLRP3) inflammasome signaling pathways. This induces excessive production of NO, TNF-α, and Interleukin-1 beta (IL-1β) in cardiomyocytes, ultimately leading to contractile dysfunction and cell death (Xia et al., 2023). During this process, a vicious cycle of oxidative stress and endoplasmic reticulum (ER) stress further exacerbates myocardial injury—manifested by GSH depletion, MDA accumulation, and suppressed SOD activity (Cao et al., 2022). Although H_2_S demonstrates considerable therapeutic potential for myocardial protection through the mechanism illustrated in Figure 2, research on its application in septic myocardial injury remains limited. Existing studies predominantly focus on ischemia-reperfusion models. Traditional H_2_S donors (e.g., Na_2_S), due to their burst-release characteristics, cause significant concentration fluctuations and are unsuitable for meeting the sustained demands of the septic disease course (Wang, 2012). Therefore, developing novel donors with controllable release kinetics, enabling multi-target regulation simultaneously addressing cardiomyocyte redox balance (GSH/MDA) and inflammatory pathways, represents a promising strategy to overcome the therapeutic challenges in septic myocardial injury.

Based on the aforementioned research background and building on previous studies, this study synthesized a series of H_2_S donors featuring a phenylphosphonodithioate core structure, with the aim of overcoming the limitations of existing H_2_S donors. This breakthrough lays a solid foundation for the clinical translation of H_2_S-based therapeutics.

## 2 Results and discussion

### 2.1 Chemical synthesis

As shown in **Route 1,** Starting from benzene, compounds **3a, 3b and 3c** andwere synthesized as hydrogen sulfide (H_2_S) donors according to a previously reported method. Compounds **3a** and **3b** have been reported in the literature, while the **3c** series remains undocumented (Xu et al., 2025; Zhang et al., 2019). Under AlCl_3_ catalysis, PCl_3_ is activated by Lewis acid to form the electrophile [Cl_3_P^+^] which attacks the benzene ring, proceeding through a σ-complex intermediate. Subsequent chlorine rearrangement of the transient intermediate phenylphosphorus trichloride (C_6_H_5_PCl_3_) yields phenyldichlorophosphine (**1**). Phenyldichlorophosphine (C_6_H_5_PCl_2_) then undergoes nucleophilic addition-elimination with elemental sulfur (S_8_) to form phenylthiophosphonyl dichloride (**2**). It is worth noting that sulfur-containing compounds are useful tools in medicinal chemistry (Akhlaghinia and Makarem, 2011; Morrison and Boyd, 2002). The reaction of phenylthiophosphonyl dichloride (C_6_H_5_P(S)Cl_2_) with nucleophiles (ethanolamine/2-amino-4-nitrophenol/diethylamine) follows an SN_2_ mechanism: nucleophilic groups (-NH_2_, -O^-^) attack the phosphorus atom to displace chlorine, affording C_6_H_5_P(S) (Nu)_2_-type products (**3**). The aromatic proton signals of the final product 3a (δ 7.30-8.80 ppm) aligned with literature data (Zhang et al., 2019), whereas the aromatic protons of donor 3b resonated at δ 8.00-7.30 ppm, exhibiting an upfield shift of 0.5 ppm relative to its precursor. This shift confirms that increased electron density on the phenyl ring attenuates deshielding effects. Donor 3c displayed analogous behavior. In summary, this study employed phenylthiophosphonyl dichloride (C_6_H_5_P(S)Cl_2_) as the key intermediate to efficiently construct a series of H_2_S donors (C_6_H_5_P(S) (Nu)_2_, 30%–45% yield) via SN_2_ nucleophilic substitution under weakly alkaline conditions (Et_3_N) in dichloromethane.

### 2.2 The toxicity of the compounds

Drug safety is a prerequisite for subsequent experiments. We first evaluated the cytotoxicity of the hydrogen sulfide (H_2_S) donor compounds against normal liver cells (LO2) and WI38 (normal human lung fibroblasts) using the MTT assay (Xu et al., 2025). LO2 and WI38 cells were seeded in 96-well plates at a density of 1 × 10^5^ cells/well. Cell viability was measured 24 h after compound treatment, with untreated cells serving as the control. The experimental results (Table 1) demonstrated that: The H_2_S donor compounds exhibited no significant cytotoxicity against LO2 (normal hepatocytes) or WI38 (normal lung fibroblasts) within the tested concentration range. The toxicity of the test compound is much lower than that of the positive control drug 5-Fu. This indicates their low potential risk to normal liver tissue and non-target organs, providing a safety basis for further development. Regarding the rationale for using 5-FU in the LO2/WI38 models, our reasons are as follows: 5-Fluorouracil (5-Fu) is a classic antitumor chemotherapeutic drug with a long history of clinical application and a well-established mechanism of action. In *in vitro* cytotoxicity assays, 5-Fu is widely accepted and established as a standard positive control compound. 5-FU is recommended as a positive control agent by ISO 10993-5 (Biological evaluation of medical devices—Part 5: Tests for *in vitro* cytotoxicity), ensuring the reliability of cytotoxicity assessment results. This provides crucial historical reference benchmarks and comparability for experimental results. The inclusion of 5-Fu as a positive control serves as a vital means of monitoring experimental reproducibility and reliability.

**TABLE 1:** IC_50_ (μM)^a^ values of all the compounds.

Compounds	LO2	WI38
**3a-1**	>200	>200
**3a-2**	>200	>200
**3a-3**	>200	>200
**3b-1**	>200	>200
**3b-2**	>200	>200
**3b-3**	>200	>200
**3c-1**	>200	>200
**3c-2**	>200	>200
**3c-3**	>200	>200
5-FU^b^	180	210

^a^IC_50_ is the minimum concentration ofa drug that is toxic to 50% of the cells. Each experiment was repeated three times.

^b^5−Fluorouracil (5−FU) is an antimetabolite chemotherapy drug.

### 2.3 H_2_S release ability of the compound and its influencing factors

**FIGURE 3:**
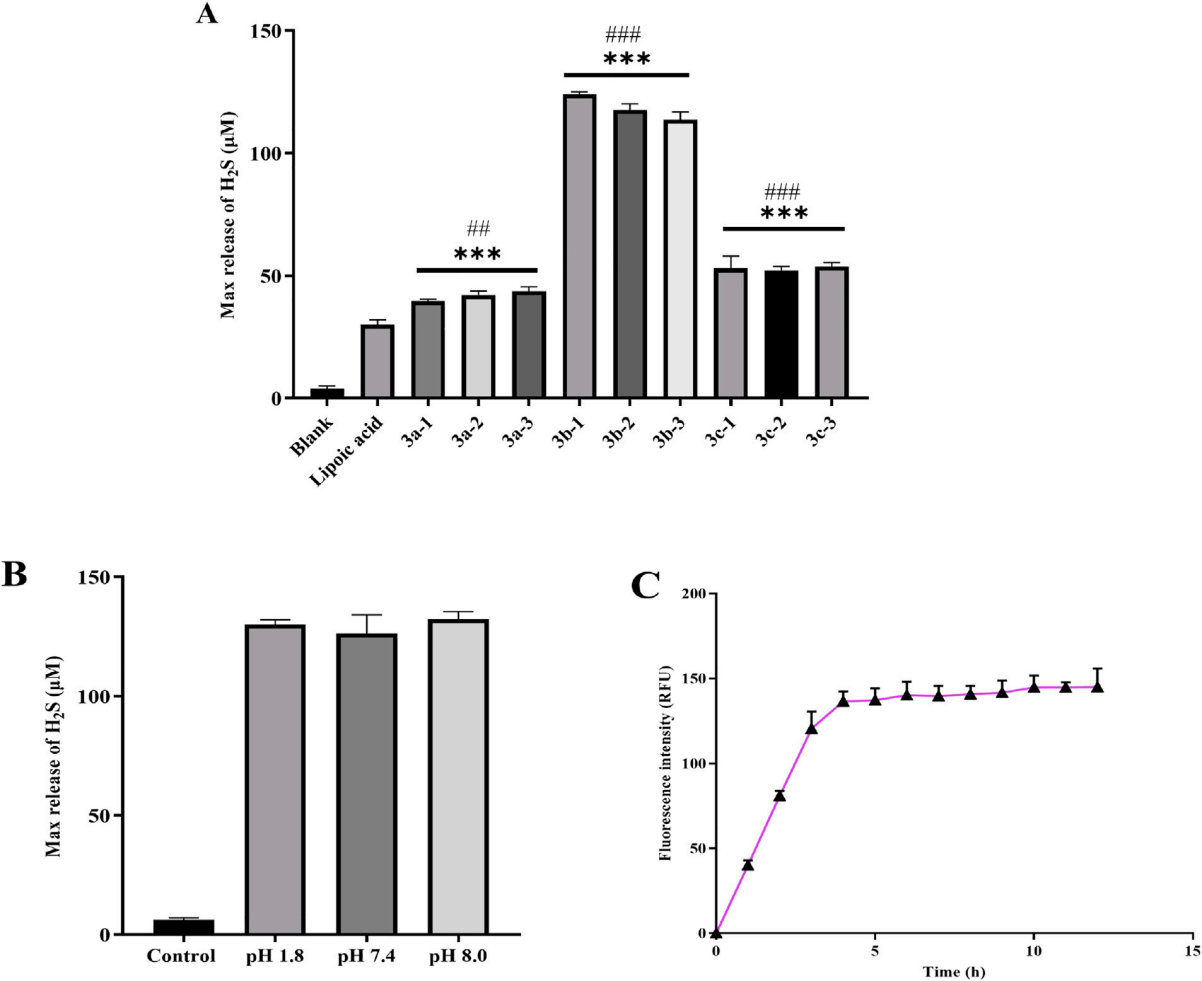
**(A)** Total H_2_S release by each compound over 6 h. **(B)** Total H_2_S release from Compound 3b-1 over 6 h under varying pH conditions. **(C)** The **H**_**2**_ **S** amount released from **3b-1** within 12 h. Compared with the Blank group, *p < 0.05, **p < 0.01, ***p < 0.001; ^#^p < 0.05, ^##^p < 0.01, ^###^p < 0.001 vs. Lipoic acid group. Data are presented as means ± SEM from three independent experiments.

Subsequently, we systematically evaluated the H_2_S release profiles of the novel hydrogen sulfide (H_2_S) donor drug under simulated physiological conditions *in vitro*. The results revealed that the compound exhibited favorable sustained-release characteristics in PBS buffer (pH 7.4) at 37 °C (Feng et al., 2015; Zhang et al., 2019). As shown in Figure 3A, the 3b series compounds demonstrated higher cumulative H_2_S release. This enhanced release efficiency is likely attributed to the stronger electron-donating system inherent to the sulfur-phosphorus core-based H_2_S donors. The pH values vary across different human tissues and organs. For instance, the pH in the small intestine is approximately 8.0, whereas that of gastric fluid is as low as approximately 1.8. Therefore, we examined the hydrogen sulfide (H_2_S) release profile of compound **3b-1**—which exhibits the strongest H_2_S-releasing capacity—under various pH conditions and its time-dependent release kinetics. As shown in Figure 3B, varying pH conditions had minimal impact on the H_2_S-releasing capacity of **3b-1**. Time-course analysis (Figure 3C) revealed that H_2_S release increased progressively over the first 5 h and subsequently reached a plateau.

**FIGURE 4:**
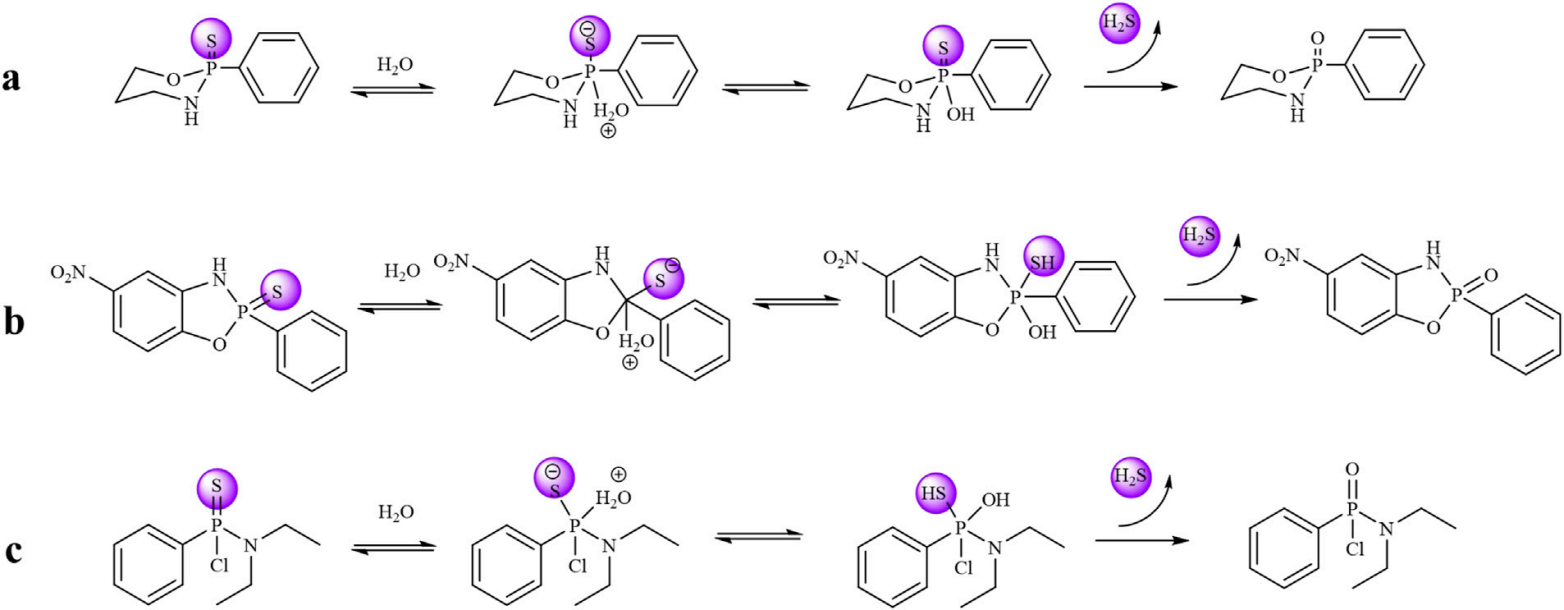
**(a)** Mechanism of compound **3a** releasing hydrogen sulfide. **(b)** Mechanism of compound **3b** releasing hydrogen sulfide. **(c)** Mechanism of compound **3c** releasing hydrogen sulfide.

The H_2_S-releasing capacity of compounds is closely associated with their molecular structures. Specifically, the **3a** series exhibits high stability, resulting in slow release kinetics and low H_2_S yield in PBS. Owing to the electron-donating effect of the benzene ring, the **3b** series demonstrates facilitated H_2_S release. In contrast, H_2_S liberation from the **3c** series is inhibited by the electron-withdrawing effect of the chlorine substituent. Based on these structure-activity relationships and supported by literature evidence, we propose the H_2_S release mechanism illustrated in Figure 4. Finally, we performed stability testing on compound **3b-1**. As shown in Supplementary Figure S1, the detection results also provide additional support for the findings in Figure 3C, indicating that the compound decomposes within 5 h and subsequently plateaus.

### 2.4 The anti-inflammatory activity of the compounds

**FIGURE 5:**
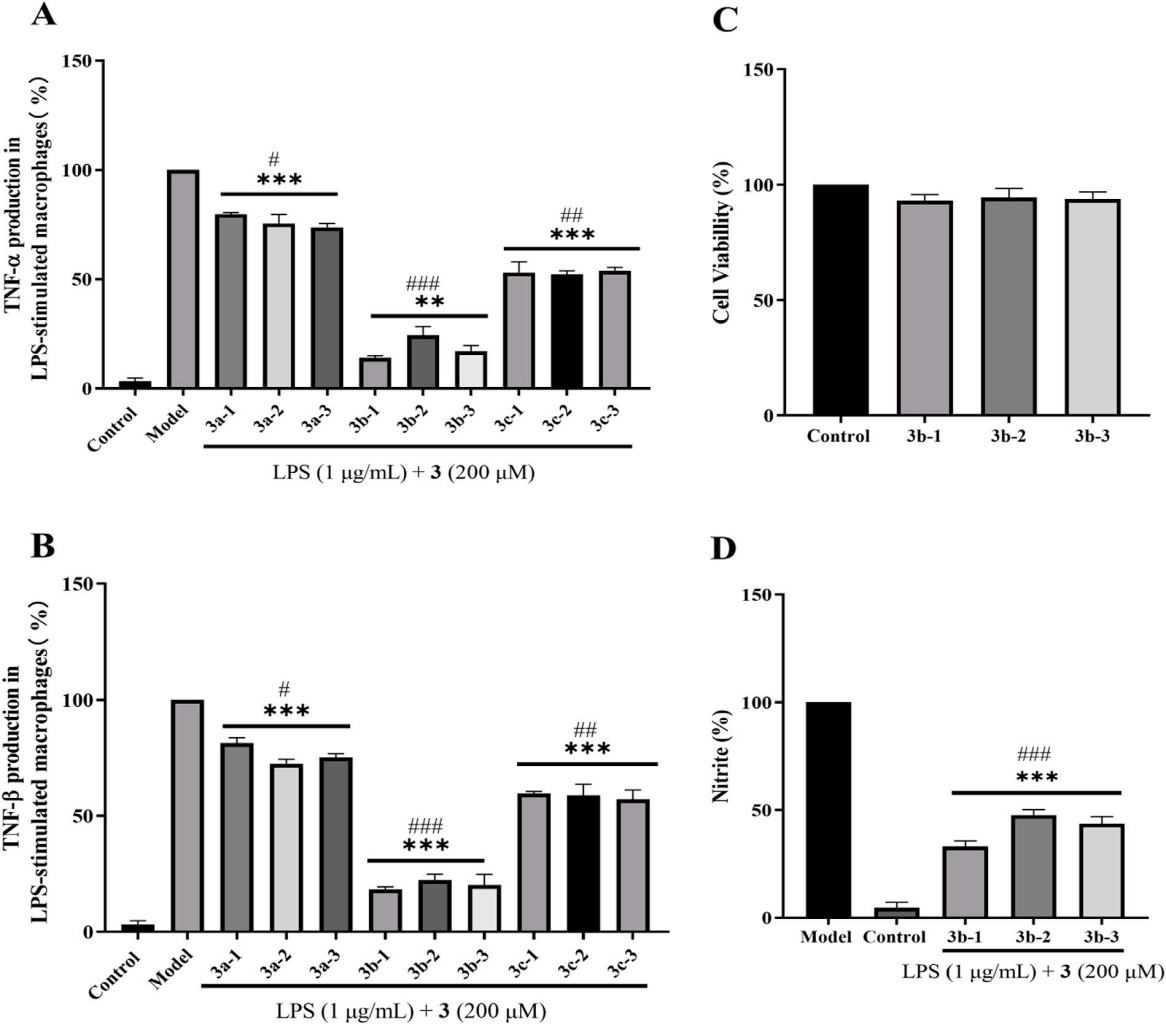
Evaluation of anti-inflammatory properties in LPS-stimulated RAW 264.7 macrophages treated with H_2_S donor compounds (200 μM). **(A)** Inhibition of pro-inflammatory cytokine TNF-α release by H_2_S donors. **(B)** Effects of H_2_S donors on RAW 264.7 macrophage viability. **(C)** Suppression of pro-inflammatory cytokine TNF-β production by H_2_S donors. **(D)** Attenuation of nitrite accumulation by H_2_S donors. Compared with the control group, *p < 0.05, **p < 0.01, ***p < 0.001; #p < 0.05, ##p < 0.01, ###p < 0.001 vs. LPS model group. Data are presented as means ± SEM from three independent experiments.

Initial screening of the nine newly synthesized compounds for anti-inflammatory activity was performed using LPS (1 μg/mL)-stimulated RAW264.7 macrophages over 24 h (Huang et al., 2016). As established, sustained inflammation drives hepatic pathology via Kupffer cell activation and pro-inflammatory cytokine cascades (e.g., TNF-α/IL-6), directly compromising hepatocyte viability. Similarly, septic cardiomyopathy involves TLR4-mediated hyperinflammation where cytokine storms (notably TNF-α, IL-1β) induce cardiomyocyte apoptosis and contractile dysfunction (Lu and Wen, 2025). Crucially, H_2_S confers organ protection through: (i) Suppression of NF-κB nuclear translocation and downstream pro-inflammatory gene expression; (ii) Modulation of inflammasome activity via inhibition of NLRP3 activation and caspase-1-dependent IL-1β maturation; (iii) Maintenance of redox homeostasis by attenuating inflammation-amplified oxidative stress through SOD/GSH pathways (Cirino et al., 2023). As shown in Figures 5A,B, the **3b**-series compounds demonstrated superior suppression of pro-inflammatory cytokines, with compound **3b-1** exhibiting the most potent activity. Subsequent MTT assays confirmed low cytotoxicity for the **3b**-series, with >95% cell viability maintained at 200 μg/mL (Figure 5C). Further evaluation of nitrite production (a marker of NO-mediated inflammation) revealed that **3b-1** most effectively attenuated inflammatory responses (Figure 5D). Given its highest H_2_S release capacity and superior anti-inflammatory efficacy, compound **3b-1** was selected as the lead candidate for subsequent mechanistic and *in vivo* studies.

### 2.5 Hepatoprotective effects

**FIGURE 6:**
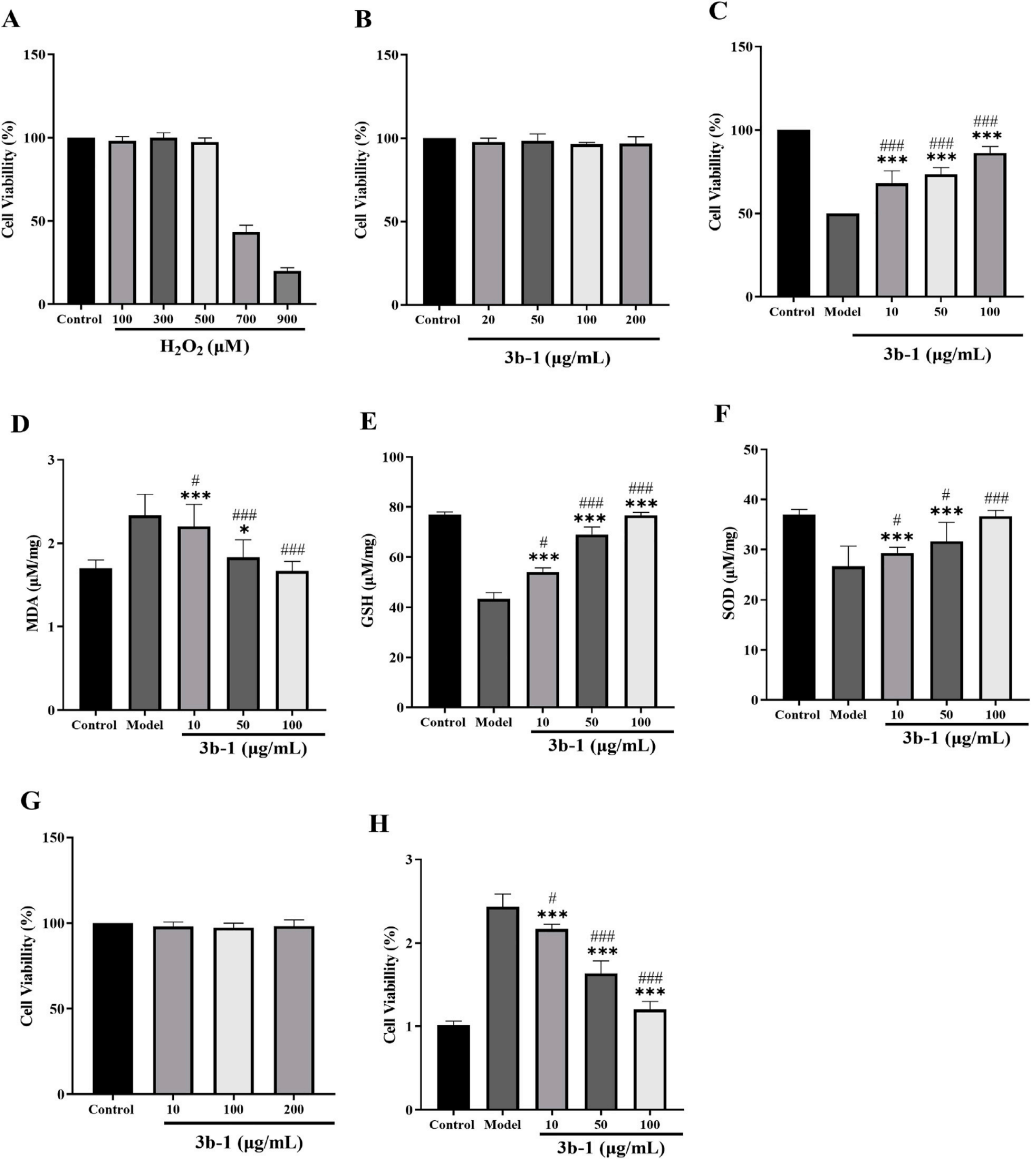
**(A)** Effect of H_2_O_2_ on the viability of BRL cells. **(B)** Effects of **3b-1** on the viability of BRL cells. **(C)** Effect of **3b-1** on restoring the cell growth in BRL cells exposed to H_2_O_2_. **(D)** Effect of **3b-1** on MDA in H_2_O_2_-treated BRL cells. **(E)** Effect of **3b-1** on GSH in H_2_O_2_-treated BRL cells. **(F)** Effect of **3b-1** on SOD in H_2_O_2_-treated BRL cells. **(G)** Effect of **3b-1** on the viability of HSC-T6 cells. **(H)** Effect of **3b-1** on the proliferation of HSC-T6 cells activated by TGF-β1. Compared with the control group, ^*^p < 0.05, ^**^p < 0.01, ^***^p < 0.001; ^#^p < 0.05, ^##^p < 0.01, ^###^p < 0.001 vs. LPS model group. Data are presented as means ± SEM from three independent experiments.

Chronic liver injury leads to excessive deposition of extracellular matrix (ECM), driving the pathogenesis of liver diseases including fibrosis, cirrhosis, and hepatocellular carcinoma (Tacke and Zimmermann, 2014). Given the recognized antioxidant properties of hydrogen sulfide donors and their potential protective effects against hepatic disorders, we evaluated the cytoprotective activity of **3b-1** in both BRL-3A rat hepatocytes and HSC-T6 immortalized murine hepatic stellate cells. A cellular model of oxidative injury was established by treating BRL-3A cells with hydrogen peroxide (H_2_O_2_, 700 μM) for 24 h. As shown in (Figure 6A, H_2_O_2_ exposure significantly reduced cell viability compared to untreated controls. Pretreatment with 200 μM **3b-1** alone exhibited no cytotoxicity (Figure 6B). **Compound 3b-1** dose-dependently restored viability in oxidatively damaged cells ((Figure 6C). Furthermore, **3b-1** treatment significantly reduced intracellular malondialdehyde (MDA) levels (p < 0.05) while enhancing superoxide dismutase (SOD) activity and glutathione (GSH) content compared to the H_2_O_2_-injured group ((Figures 6D–F). These data demonstrate that **3b-1** protects hepatocytes against oxidative stress-induced damage. Hepatic fibrosis represents a wound-healing response characterized by ECM accumulation following chronic injury, with hepatic stellate cells (HSCs) serving as the primary ECM-producing cells (Roehlen et al., 2020). We thus assessed the anti-fibrotic potential of **3b-1** by examining its effects on TGF-β1-activated HSC-T6 cells. **Compound 3b-1** dose-dependently suppressed HSC-T6 proliferation (Figures 6G,H). Collectively, **3b-1** protects against oxidative liver injury through reducing lipid peroxidation (MDA), enhancing antioxidant capacity (SOD, GSH), and inhibiting HSC activation. These dual mechanisms position **3b-1** as a promising therapeutic candidate for liver injury and fibrosis.

### 2.6 Cardioprotective effects

In recent years, sepsis in its early stages has been recognized as a clinical syndrome characterized by a systemic inflammatory response syndrome (SIRS) to infection. The heart is one of the organs most vulnerable to injury and dysfunction in sepsis, and sepsis-induced myocardial injury is relatively common in the ICU (Srzić et al., 2022). Previous studies have demonstrated that hydrogen sulfide (H_2_S) ameliorates cardiac dysfunction in septic rats by reducing cardiomyocyte injury caused by excessive autophagy through the activation of the AMPK/mTOR pathway (Yang et al., 2017).

**FIGURE 7:**
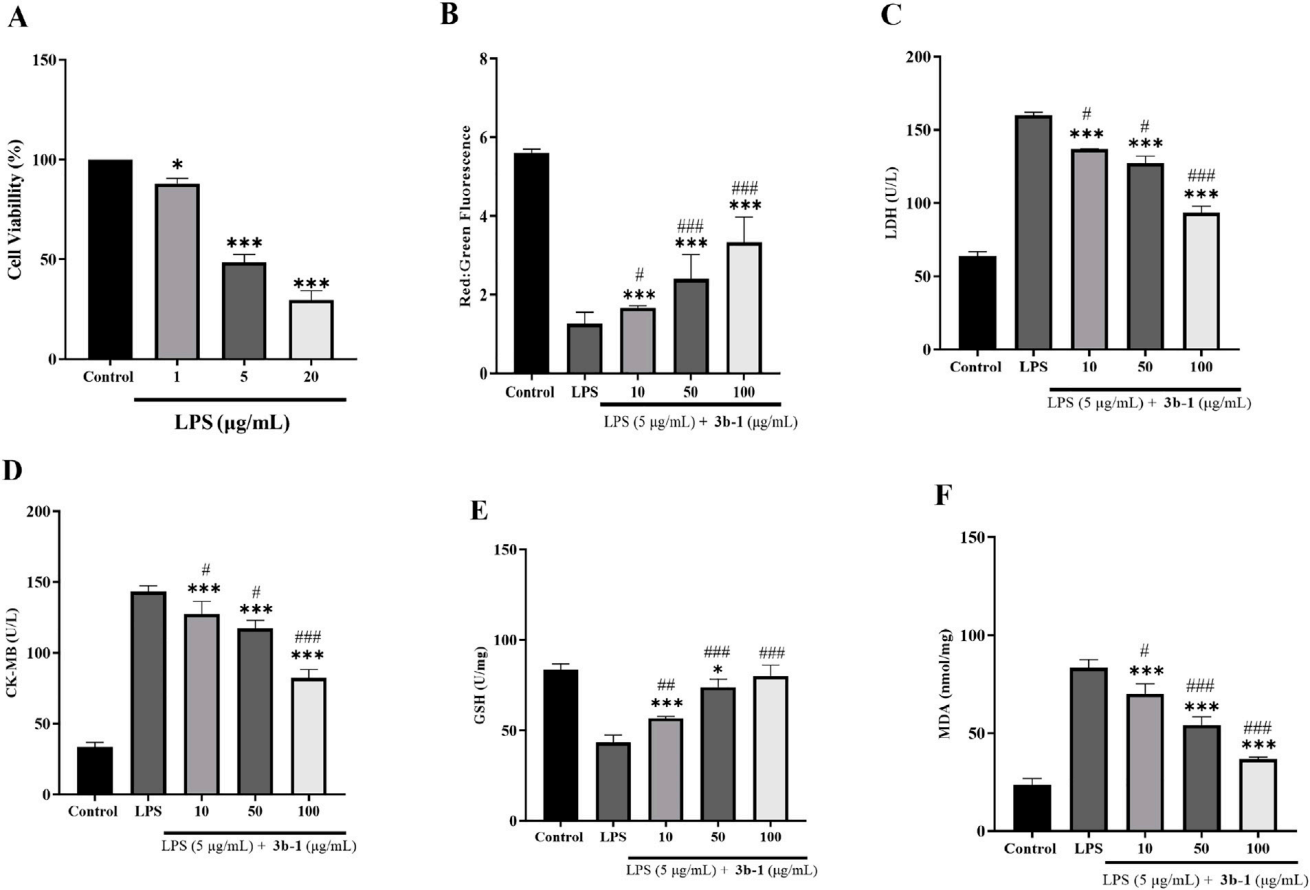
**(A)** Effect of varying LPS concentrations on H9c2 cell viability. **(B)** Impact of **3b-1** on mitochondrial membrane potential in LPS-stimulated cells. **(C)** Effect of **3b-1** on LDH release in LPS-treated cells. **(D)** Influence of **3b-1** on CK-MB levels in LPS-induced cells. **(E)** Effect of **3b-1** on GSH activity in LPS-stimulated cells. **(F)** Impact of **3b-1** on MDA production in LPS-exposed cells. Compared with the control group, ^*^p < 0.05, ^**^p < 0.01, ^***^p < 0.001; ^#^p < 0.05, ^##^p < 0.01, ^###^p < 0.001 vs. LPS model group. Data are presented as means ± SEM from three independent experiments.

To assess the cardioprotective capacity of compound **3b-1**, we established an *in vitro* model of septic cardiomyopathy using lipopolysaccharide (LPS)-challenged H9c2 cardiomyocytes. Initial dose-response analysis via CCK-8 assay (Figure 7A) demonstrated concentration-dependent cytotoxicity: 1 μg/mL LPS: Modest viability reduction *versus* control, 5 μg/mL LPS: Significant 51.6% viability decrease, 20 μg/mL LPS: Severe viability impairment. The intermediate LPS concentration (5 μg/mL) was selected for subsequent assays to maintain measurable pathological responses while ensuring experimental feasibility. To evaluate the protective effects of **3b-1**, JC-1 probe analysis (Figure 7B) demonstrated that **3b-1** treatment effectively attenuated LPS-induced mitochondrial membrane potential disruption in cardiomyocytes. Subsequent measurements of cardiac injury markers (Figures 7C,D) showed that LPS stimulation significantly increased Lactate Dehydrogenase (LDH) and Creatine Kinase-Myocardial Band (CK-MB) release compared to the control group (P < 0.05), while **3b-1** administration significantly reduced these elevations (P < 0.05). Assessment of oxidative stress parameters (Figures 7E,F) revealed that LPS exposure significantly decreased GSH activity and increased MDA content relative to control (P < 0.05). Importantly, **3b-1** intervention restored GSH activity and reduced MDA levels (P < 0.05). These findings collectively demonstrate that the H_2_S donor **3b-1** effectively mitigates LPS-induced cardiomyocyte injury through preservation of mitochondrial function, reduction of cardiac injury markers, and attenuation of oxidative stress.

## 3 Conclusion

Building on previously reported compounds, this study synthesized a series of H_2_S-donor derivatives based on the phenylphosphonothioic dichloride scaffold using established synthetic methodologies. Among them, compound **3b-1** was identified as a highly efficient H_2_S donor and demonstrated significant *in vitro* anti-inflammatory activity, effectively reducing the levels of key inflammatory cytokines TNF-α, TNF-β, and nitrite. In an H_2_O_2_-induced oxidative injury model using BRL hepatocytes, **3b-1** exerted potent hepatoprotective effects by reducing malondialdehyde (MDA) production, enhancing the activities of superoxide dismutase (SOD) and glutathione (GSH), and inhibiting the activation of hepatic stellate cells (HSCs), collectively protecting against oxidative liver damage. This indicates its potential as a candidate for treating oxidative liver injury and liver fibrosis. Concurrently, in an LPS-induced cardiomyocyte injury model (mimicking septic cardiomyopathy), **3b-1** confirmed its distinct cardioprotective efficacy by preserving mitochondrial function, reducing the release of cardiac injury markers, and alleviating oxidative stress. Collectively, these findings demonstrate that **3b-1** is a multifunctional candidate molecule integrating efficient H_2_S release, potent anti-inflammatory, antioxidant, hepatoprotective, and cardioprotective activities, providing a significant foundation for developing novel therapeutic strategies against related diseases such as liver injury, liver fibrosis, and septic cardiomyopathy.

## 4 Experimental section

### 4.1 Chemically synthetical experiments

All chemicals were of reagent grade or higher purity, purchased from Adamas and used directly without further purification. Solvents were used as received or dried over molecular sieves as appropriate. Column chromatography was performed using silica gel (100–200 mesh, Qingdao Ocean Chemical Factory), with reaction progress monitored by TLC (silica gel GF254 plates, Yantai Jiangyou Silica Gel Development Co., Ltd). All key intermediates and final products were characterized by ^1^H NMR (400 MHz) and ^13^C NMR (100 MHz) spectra recorded on a Bruker Avance 400 spectrometer. Chemical shifts are reported in ppm using residual solvent peaks as internal references (CDCl_3_: δ 7.26 ppm for ^1^H NMR, δ 77.16 ppm for ^13^C NMR). Products were further characterized by high-resolution electrospray ionization mass spectrometry (ESI-HRMS) using an AB Sciex TripleTOF 5600+ mass spectrometer.

#### 4.1.1 Dichloro(phenyl)phosphane (**1**)

Benzene (0.1 mmol), phosphorus trichloride (0.3 mmol), and aluminum chloride (0.12 mmol) were combined in a reaction flask and stirred under reflux for 5 h. Phosphorus trichloride (0.12 mol) and petroleum ether (45 mL) were then added, and stirring continued under reflux for 30 min. After cooling to room temperature, the mixture was filtered under reduced pressure. The filtrate was distilled at atmospheric pressure using petroleum ether, followed by distillation under reduced pressure. The fraction distilling at 100 °C was collected.

Yield, 75%. ^1^H NMR (400 MHz, Chloroform-*d*) δ 7.94 (t, *J* = 8.6 Hz, 2H), 7.61–7.51 (m, 3H). ^13^C NMR (101 MHz, Chloroform-d) δ 140.72 (d, *J*=52.2 Hz), 132.79, 130.33 (d, *J*=31.3 Hz), 129.04 (d, *J*=7.9 Hz). TOF-MS, m/z: [M + H]^+^, calcd. for C_6_H_6_Cl_2_P^+^, 178.9506, found: 178.9511.

#### 4.1.2 Phenylphosphonothioic dichloride (**2**)

Sulfur powder was added slowly in batches to the previous product (**1**) at 30 °C. Once most sulfur had dissolved and the solution became viscous, the temperature was raised to 80 °C and stirring continued for 1 h. Unreacted phosphorus trichloride and benzene were removed by distillation under atmospheric pressure followed by vacuum distillation. The fraction distilling at 90 °C was collected as phenyl phosphorothioic dichloride.

Yield, 40%. ^1^H NMR (400 MHz, Chloroform-*d*) δ 8.13 (dd, *J* = 18.6, 7.9 Hz, 2H), 7.63 (dt, *J* = 6.0, 3.0 Hz, 1H), 7.60–7.50 (m, 2H). ^13^C NMR (101 MHz, Chloroform-d) δ 138.71 (d, *J* = 118.2 Hz), 133.87 (d, *J* = 4.0 Hz), 130.1 (d, *J* = 15.2 Hz), 128.85 (d, *J* = 18.2 Hz). TOF-MS, m/z: [M + H]+, calcd. for C_6_H_6_Cl_2_SP^+^, 210.9227, found: 210.9237.

#### 4.1.3 2-phenyl-1,3,2-oxazaphospholidine 2-sulfide (**3a-1**)

2-Aminoethanol (2 mmol) and triethylamine (1 mL) were dissolved in dichloromethane. Phenylphosphonous dichloride (PhPCl_2_, 2 mmol) was added dropwise to the solution in an ice bath. The mixture was stirred at room temperature for 8 h. The resulting precipitate was filtered off, and the filtrate was concentrated. The crude product was purified by column chromatography (PE/EA = 4:1) to afford a white solid.

Yield, 42%. 1H NMR (400 MHz, Chloroform-d) δ 7.95–7.75 (m, 2H), 7.53–7.33 (m, 3H), 4.52–4.46 (m, 1H), 4.37–4.28 (m, 1H), 3.75–3.66 (m, 1H), 3.48–3.41 (m, 1H), 3.20–3.01 (m, *J* = 19 Hz, 1H). ^13^C NMR (101 MHz, Chloroform-d) δ 136.47 (d, *J* = 135.0 Hz), 132.15 (d, *J* = 3.2 Hz), 130.96 (d, *J* = 12.4 Hz), 128.49 (d, *J* = 14.8 Hz), 77.16, 68.35, 43.97. IR (KBr, cm^-1^): 3265 (NH), 1436 (P=S), 1121 (C-O). TOF-MS, m/z: [M + H]^+^, calcd. for C_8_H_11_NOPS^+^, 200.0299, found: 200.0303.

#### 4.1.4 2-phenyl-1,3,2-oxazaphosphinane 2-sulfide (**3a-2**)

Yield, 45%. ^1^H NMR (400 MHz, Chloroform-*d*) δ 7.86–7.81 (m, *J* = 6.7, 2H), 7.50–7.48 (m, *J* = 4.8 Hz, 3H), 4.50–4.41 (m, 1H), 4.10–4.02 (m, *J* = 6.4 Hz, 1H), 3.46–3.13 (m, 3H), 2.10–2.02 (m, 1H), 1.62 (d, *J* = 14.3 Hz, 1H). ^13^C NMR (101 MHz, Chloroform-d) δ 134.34 (d, *J* = 133.7 Hz), 131.92 (d, *J* = 3.2 Hz), 130.82 (d, *J* = 11.3 Hz), 129.03 (d, *J* = 14.1 Hz), 67.84, 41.33, 26.70. IR (KBr, cm^-1^): 3274 (NH), 1438 (P=S), 1120 (C-O). TOF-MS, m/z: [M + H]^+^, calcd. for C_9_H_13_NOPS^+^, 214.0455, found: 214.0462.

#### 4.1.5 2-phenyl-1,3,2-oxazaphosphepane 2-sulfide (**3a-3**)

Yield, 50%. ^1^H NMR (400 MHz, Chloroform-*d*) δ 7.92–7.81 (m, 2H), 7.48–7.39 (m, *J* = 2.7, 3H), 4.56 (m, 1H), 4.17 (m, *J* = 13.1, 1H), 3.46 (s, 1H), 3.16–3.03 (m, *J* = 4.7 Hz, 1H), 2.80–2.72 (m, *J* = 8 Hz, 1H), 1.97–1.69 (m, 3H), 1.64–1.47 (m, 1H). ^13^C NMR (101 MHz, Chloroform-*d*) δ 135.13 (d, *J* = 148.6 Hz), 131.41 (d, *J* = 3.2 Hz), 130.32 (d, *J* = 11.0 Hz), 128.37 (d, *J* = 14.5 Hz), 64.95, 42.65, 31.69, 29.70. IR (KBr, cm^-1^): 3436 (NH), 1438 (P=S), 1161 (C-O). TOF-MS, m/z: [M + H]^+^, calcd. for C_10_H_15_NOPS^+^, 228.0612, found: 228.0617.

#### 4.1.6 Phenylphosphonothioic dichloride (**3b-1**)

Yield, 40%. ^1^H NMR (400 MHz, DMSO-*d*_6_) δ 8.24–8.18 (m, 1H), 7.89 (d, *J* = 7.1 Hz, 2H), 7.79–7.75 (m, 1H), 7.69–7.64 (m, 1H), 6.84 (d, *J* = 12 Hz, 1H), 6.68 (s, 3H). ^13^C NMR (101 MHz, DMSO-*d*_6_) δ 147.68, 134.81 (d, *J* = 7.7 Hz), 134.12 (d, *J* = 3.1 Hz), 131.72 (d, *J* = 12.9 Hz), 128.91 (d, *J* = 15.6 Hz), 123.13, 117.18 (d, *J* = 4.0 Hz), 113.98. IR (KBr, cm^-1^): 3260 (NH), 1435 (P=S). ^31^P NMR (162 MHz, DMSO) 169.50. TOF-MS, m/z: [M + H]^+^, calcd. For C_12_H_10_N_2_O_3_PS^+^, 293.0150, found: 293.0148.

#### 4.1.7 5-methyl-2-phenyl-3H benzo[*d*][1,3,2]oxazaphosphole 2-sulfide (**3b-2**)

Yield, 38%. 1H NMR (400 MHz, Chloroform-*d*) δ 7.85–7.50 (m, 2H), 7.50–7.45 (m, 1H), 7.42–7.37 (m, 2H), 6.68 (d, *J* = 8.0 Hz, 2H), 6.46 (d, *J* = 8.0 Hz, 1H), 5.82 (d, *J* = 12 Hz, 1H), 2.17 (s, 3H). ^13^C NMR (101 MHz, Chloroform-*d*) δ 146.28 (d, *J* = 7.4 Hz), 146.20, 133.84, 133.75, 132.16 (d, *J* = 3.2 Hz), 132.13, 130.98 (d, *J* = 11.4 Hz), 130.87, 128.78 (d, *J* = 14.8 Hz), 124.83, 124.80, 121.53, 120.18 (d, *J* = 2.9 Hz), 116.40, 20.89. IR (KBr, cm^-1^): 3265 (NH), 1435 (P=S). TOF−MS, m/z: [M+H]+, calcd. for C_13_H_13_NOPS+, 262.0455, found: 262.0459.

#### 4.1.8 2-phenyl-3H-benzo[d][1,3,2]oxazaphosphole-5-carbonitrile 2-sulfide (**3b-3**)

Yield, 41%. 1H NMR (400 MHz, Chloroform-*d*) δ 7.86–7.80 (m, 2H), 7.49–7.37 (m, 1H), 7.42–7.37 (m, 2H), 6.84–6.79 (m, 2H), 6.67–6.63 (m, 1H), 5.99 (d, *J* = 12.0 Hz, 1H). ^13^C NMR (101 MHz, Chloroform-*d*) δ 145.65 (d, *J* = 7.8 Hz), 133.85, 132.18 (d, *J* = 3.2 Hz), 130.88 (d, *J* = 11.5 Hz), 128.76 (d, *J* = 14.8 Hz), 127.91 (d, *J* = 3.1 Hz), 123.23, 121.14, 119.40 (d, *J* = 3.2 Hz), 115.58. IR (KBr, cm^-1^): 3265 (NH), 1433 (P=S). TOF−MS, m/z: [M+H]+, calcd. for C_13_H_10_N_2_OPS+, 273.0251, found: 273.0255.

#### 4.1.9 Phenylphosphonothioic dichloride (**3c-1**)

Yield, 43%. 1H NMR (400 MHz, Chloroform-*d*) δ 7.92–7.86 (m, 2H), 7.54–7.34 (m, 3H), 3.38–3.14 (m, 4H), 1.08 (t, *J* = 7.1 Hz, 6H). ^13^C NMR (101 MHz, Chloroform-*d*) δ 136.49 (d, *J* = 136.1 Hz), 132.02 (d, *J* = 3.6 Hz), 130.14 (d, *J* = 12.1 Hz), 128.53 (d, *J* = 15.8 Hz), 40.33 (d, *J* = 3.0 Hz), 13.02 (d, *J* = 6.0 Hz). IR (KBr, cm^-1^): 1435 (P=S). TOF-MS, m/z: [M + H]^+^, calcd. For C_10_H_16_ClNPS^+^, 248.0429, found: 248.0433. The observed isotopic pattern is consistent with the theoretical distribution: experimental ratio [M + H]^+^: [M + H+2]^+^ = 3 : 1.

#### 4.1.10 Phenylphosphonothioic dichloride (**3c-2**)

Yield, 45%. 1H NMR (400 MHz, Chloroform-*d*) δ 7.96–7.90 (m, 2H), 7.48 (m, 3H), 3.28–3.06 (m, 4H), 1.60–1.52 (m, 2H), 1.47–1.39 (m, 2H), 1.25–1.15 (m, 4H), 0.83 (t, *J* = 7.4 Hz, 6H). ^13^C NMR (101 MHz, Chloroform-d) δ 137.27 (d, *J* = 136.0 Hz), 132.12 (d, *J* = 3.5 Hz), 130.58 (d, *J* = 12.0 Hz), 128.58 (d, *J* = 15.8 Hz), 46.10 (d, *J* = 2.0 Hz), 29.79 (d, *J* = 5.0 Hz), 20.03 (d, *J* = 2.0 Hz), 13.76 (d, *J* = 2.0 Hz). IR (KBr, cm^-1^): 1435 (P=S). TOF-MS, m/z: [M + H]^+^, calcd. For C_14_H_24_ClNPS^+^, 304.1055, found: 304.1059. The observed isotopic pattern is consistent with the theoretical distribution: experimental ratio [M + H]^+^: [M + H+2]^+^ = 3 : 1.

#### 4.1.11 Phenylphosphonothioic dichloride (**3c-3**)

Yield, 45%. 1H NMR (400 MHz, Chloroform-*d*) δ 7.95–7.89 (m, 2H), 7.55–7.46 (m, 3H), 3.67 (t, *J* = 4.7 Hz, 4H), 3.44–3.36 (m, 2H), 3.08–3.00 (m, 2H). ^13^C NMR (101 MHz, Chloroform-*d*) δ 134.70 (d, *J* = 134.3 Hz), 132.01 (d, *J* = 3.0 Hz), 130.57 (d, *J* = 11.9 Hz), 128.90 (d, *J* = 15.7 Hz), 66.34 (d, *J* = 9.9 Hz), 45.31 (d, *J* = 2.8 Hz). IR (KBr, cm^-1^): 1435 (P=S). TOF-MS, m/z: [M + H]^+^, calcd. For C_10_H1_4_ClNOPS^+^, 262.0222, found: 262.0225. The observed isotopic pattern is consistent with the theoretical distribution: experimental ratio [M + H]^+^: [M + H+2]^+^ = 3 : 1.

### 4.2 Cell viability

We used the CCK8 assay to detect cell viability. See Supplementary Material for more details (Xu et al., 2025).

### 4.3 H_2_S measurement

The release of hydrogen sulfide was detected by the methylene blue method. See Supplementary Material for more details (Zhao et al., 2019).

### 4.4 Treatment of compounds on LPS-Stimulated RAW264.7 macrophages

See Supplementary Material for more details (Li et al., 2023).

### 4.5 Measurement of cytokines

Culture supernatants were analyzed for cytokine levels using commercial ELISA kits (e.g., mouse TNF-α) according to the manufacturer’s protocols. Absorbance was measured at [specify wavelength] nm using a microplate reader (Dogra, 2025).

### 4.6 Superoxide dismutase (SOD) measurement

**3b-1** was determined using a commercial total SOD assay kit based on the nitroblue tetrazolium (NBT) method. See Supplementary Material for more details (Wen et al., 2022).

### 4.7 Hepatoprotective and anti-fibrotic effects of 3b-1

See Supplementary Material for more details (Dogra, 2025; He et al., 2025).

### 4.8 H9c2 cell culture

See Supplementary Material for more details (Sun et al., 2022).

### 4.9 Measurement of cardiac enzymes (LDH and CK-MB)

See Supplementary Material for more details (Zheng et al., 2022).

### 4.10 Determination of compound 3b-1 stability in PBS

See Supplementary Material for more details.

### 4.11 Statistical analysis

The above experimental data are the mean ± SD of at least three independent experiments. SPSS 22.0 software was used to process the data, and one-way analysis of variance (ANOVA) was used to measure statistical differences between the two groups.

## Data Availability

The original contributions presented in the study are included in the article/Supplementary Material, further inquiries can be directed to the corresponding author.
