# Supplementary material for: Activity evaluation of multifunctional H2S donors for anti-inflammatory, cardioprotective, and hepatoprotective applications
Source: Front Chem. 2025 Sep 5;13:1643663. doi: 10.3389/fchem.2025.1643663 (PMC12447644; doi:10.3389/fchem.2025.1643663)
Supplement: Supplementary file 1 [file DataSheet1.doc]

# **Activity Evaluation of Multifunctional H₂S Donors for Anti-Inflammatory, Cardioprotective and Hepatoprotective Applications**

Donghe Wang [[1]](#footnote-2)*, Yujie Meng ^1^, Yihong Liu ^1^

*^1^The Second Hospital of Qinhuangdao, Qinhuangdao, China, 066000*

**Table of Contents**

**H2S measurement**·············································································1

**RAW264.7** **Cell culture**······································································1

**Cell viability**···················································································2

**LPS-Stimulated RAW264.7 Macrophages** ···············································2

**Superoxide Dismutase (SOD) Measurement**············································2

**Hepatoprotective and anti-fibrotic effects of 3b-1**·····································3

**H9c2** **Cell culture**·············································································4

**Measurement of Cardiac Enzymes (LDH and CK-MB)**·····························5

**Determination of Compound 3b-1 Stability in PBS (HPLC Method)**·············5

**Spectral data**···············································································6-25

**H2S measurement**

Preparation of Na₂S Standard Solutions: A 5 mM Na₂S stock solution (prepared from Na₂S·9H₂O, 120 mg in 100 mL sodium phosphate buffer, 20 mM) was used to generate standard solutions. Aliquots (50, 100, 200, 400, 600, 800, 1000, 1500 μL) of the stock were diluted to 50 mL with sodium phosphate buffer, yielding final Na₂S concentrations of 5, 10, 20, 40, 60, 80, 100, and 150 μM.

Methylene Blue Assay & Calibration: For each standard (in triplicate), 1 mL aliquot was reacted at room temperature (≥15 min) with methylene blue reagent: 30 mM FeCl₃ in 1.2 M HCl, 20 mM N,N-dimethyl-1,4-phenylenediamine sulfate in 7.2 M HCl, and 1% w/v Zn(CH₃COO)₂ in H₂O. Absorbance was measured at 670 nm to establish the Na₂S calibration curve.

H₂S Release Measurement: The reaction was initiated by adding 75 μL of donor stock (40 mM in THF) to 30 mL phosphate buffer containing 1.0 mM TECP accelerator or L-cysteine. At timed intervals, 2.0 mL aliquots were transferred to cuvettes containing zinc acetate (1% w/v, 200 μL), N,N-dimethyl-1,4-phenylenediamine sulfate (20 mM, 400 μL in 7.2 M HCl), and FeCl₃ (30 mM, 400 μL in 1.2 M HCl). After 15 min, absorbance at 670 nm was measured. H₂S concentrations were determined using the Na₂S calibration curve.

**RAW264.7** **Cell culture**

RAW264.7 cells were cultured in Dulbecco’s Modified Eagle’s Medium (DMEM) supplemented with 10% heat-inactivated fetal bovine serum, 4 mM L-glutamine, 1 mM sodium pyruvate, 20 mM HEPES buffer, 100 U/mL penicillin, and 100 µg/mL streptomycin. Cells were maintained in 100 mm bacteriological petri dishes at 37°C under 5% CO₂ with >95% humidity. Complete medium was refreshed every two days, and subculturing was performed at 80-90% confluency using 0.25% Trypsin-EDTA digestion for 3 min at 37°C followed by neutralization with complete medium. After centrifugation at 300 × g for 5 min, cell pellets were resuspended and seeded at 5 × 10⁴ cells/cm². All cells used for experiments were between passages 5-20 and routinely confirmed mycoplasma-free using PCR detection. Cell viability was consistently >98% as determined by Trypan Blue exclusion assay prior to experimental use (Feng et al., 2016, Huang et al., 2016).

**Cell viability**

Cell viability was quantified using the Cell Counting Kit-8 (CCK-8) according to manufacturer's specifications. Briefly, RAW264.7 cells were seeded at a density of 5 × 10³ cells/well in 96-well flat-bottom microplates and allowed to adhere for 24 hr under standard culture conditions (37°C, 5% CO₂). Following treatment with compound 3b-1 or controls for 24 hr, 10 μL of CCK-8 reagent was added directly to each well containing 100 μL culture medium, avoiding air bubble formation. Plates were gently agitated on an orbital shaker (50 rpm, 1 min) to ensure homogeneous distribution and subsequently incubated for 4 hr at 37°C in a humidified 5% CO₂ atmosphere. Absorbance was measured at 450 nm using a SpectraMax M5 microplate reader (Molecular Devices) with automated pathlength correction. Viability was calculated as: Viability (%) = [(OD_treatment_ - OD_blank_) / (OD_control_ - OD_blank_)] × 100 where OD_blank_ represents wells containing medium + CCK-8 without cells, and OD_control_ represents untreated cells. All conditions were assayed in six biological replicates and validated in three independent experiments (Zhang et al., 2019, Feng et al., 2016 and Huang et al., 2016).

**LPS-Stimulated RAW264.7 Macrophages**

For LPS stimulation experiments, RAW264.7 cells were seeded at optimized densities in Nunc plates: 24-well (2.00×10⁵ cells/well in 1 mL) or 6-well (1.00×10⁶ cells/well in 2 mL) using high-glucose DMEM supplemented with 10% heat-inactivated FBS, 4 mM L-glutamine, and 1% penicillin-streptomycin. After 16-18 hr incubation at 37°C/5% CO₂, cells were washed twice with warm PBSand switched to serum-reduced medium (2% FBS). Test compounds (dissolved in DMSO with final concentration ≤0.1%) were pretreated for 30 min, followed by stimulation with ultrapure *E. coli* O111:B4 LPS (1 μg/mL in endotoxin-free water) for 6 hr. Control groups included: (1) Vehicle (0.1% DMSO), (2) LPS-only, and (3) LPS + **3b-1** (200 μM) (Huang et al., 2016).

**Superoxide Dismutase (SOD) Measurement**

**3b-1** (10, 50, 100 μg/mL) was determined using a commercial total SOD assay kit based on the nitroblue tetrazolium (NBT) method. Assay reagents were added to 96-well plates, incubated at 37°C for 30 min, and absorbance measured at 560 nm. All samples were analyzed in triplicate (He et al., 2025).

The SOD inhibition rate (%) was calculated as:

I = [(A₁ - A₂) - (A₄ - A₃)] / (A₁ - A₂) × 100

SOD activity was expressed as:

U/mg DW = (I × V₁ × N) / (50% × V₂ × W)

| Symbol | Definition |
| --- | --- |
| A₁ | Absorbance (no sample) |
| A₂ | Absorbance (no sample, no reaction starter) |
| A₃ | Absorbance (no reaction starter) |
| A₄ | Absorbance (test sample) |
| V₁ | Total reaction volume (mL) |
| V₂ | Sample volume (mL) |
| N | Sample dilution factor |
| W | Sample concentration (mg/mL) |
| DW | Dry weight |

**Hepatoprotective and anti-fibrotic effects of 3b-1**

BRL hepatocytes and HSC-T6 hepatic stellate cells were cultured in high-glucose DMEM supplemented with 1% fetal bovine serum and 1% penicillin-streptomycin at 37°C under 5% CO₂. Cells in logarithmic growth phase were seeded in 96-well plates and treated with gradient concentrations of **3b-1**(10, 50, 100 μg/mL), with cell viability assessed using CCK-8 assays. To evaluate hepatoprotective effects, BRL cells were challenged with H₂O₂ to establish an oxidative injury model, followed by assessment of **3b-1**'s impact on cell growth and biomarkers (MDA, SOD, GSH). Following a 24-hour incubation period, cells were collected into centrifuge tubes and pelleted by centrifugation. The supernatant was discarded, and the cell pellet was resuspended in 1 mL of lysing solution. The suspension underwent 2-3 freeze-thaw cycles. The resulting supernatant was collected and stored at 4°C for subsequent analysis. For measurement, 200 μL aliquots (corresponding to 2 × 10⁴ cells/mL) were transferred to a 96-well microplate, and absorbance was determined using a microplate reader. For anti-fibrotic evaluation, HSC-T6 cells were stimulated with transforming growth factor-β1 (TGF-β1) to induce fibrogenesis, with subsequent measurement of **3b-1**'s effects on proliferation in fibrotic cells (He et al., 2025).

**H9c2** **Cell culture**

H9c2 rat cardiomyocytes were cultured in high-glucose DMEM medium supplemented with 10% fetal bovine serum (FBS) and 1% penicillin-streptomycin at 37.5°C under 5% CO₂ for 24 hours until reaching >80% confluency as adherent monolayers. For subculturing, the medium was removed, and cells were washed twice with 1× PBS to eliminate dead cells and debris. Subsequently, 900 μL of trypsin solution was added to each flask to dissociate the cells; the enzymatic reaction was stopped after 1 minute by immediate addition of complete medium. The resulting cell suspension was aspirated into sterile centrifuge tubes, balanced, and centrifuged at 1500 × g for 10 minutes. The supernatant was carefully decanted to avoid disturbing the pellet, followed by resuspension in 1 mL of complete medium via gentle pipetting. For experiments, sterile 6-well plates were pre-filled with 2 mL of complete medium per well, after which 166 μL of the resuspended cell solution was added to each well. The plates were gently swirled to ensure uniform distribution and incubated at 37°C under 5% CO₂ for an additional 24 hours prior to subsequent treatments. Lipopolysaccharide (LPS) was dissolved in PBS. Following determination of the optimal LPS concentration, a stock solution of compound **3b-1** was prepared and diluted to working concentrations (10, 50, and 100 μM). Cells were pretreated with **3b-1** for 1 hour before stimulation with the optimal dose of LPS. Experimental groups included: LPS alone, LPS + 10 μM **3b-1**, LPS + 50 μM **3b-1**, and LPS + 100 μM **3b-1**. Following a 24-hour incubation period, cells were collected into centrifuge tubes and pelleted by centrifugation. The supernatant was discarded, and the cell pellet was resuspended in 1 mL of lysing solution. The suspension underwent 2-3 freeze-thaw cycles. The resulting supernatant was collected and stored at 4°C for subsequent analysis. For measurement, 200 μL aliquots (corresponding to 2 × 10⁴ cells/mL) were transferred to a 96-well microplate, and absorbance was determined using a microplate reader (Yang et al., 2017).

**Measurement of Cardiac Enzymes (LDH and CK-MB)**

H9c2 cardiomyocytes were seeded in sterile culture dishes and incubated overnight at 37°C under 5% CO₂. Cells were subsequently treated with LPS (1-20 μg/mL) and **3b-1**(10, 50, 100 μg/mL) for 24 h. Following treatment, cells were collected by pipetting into sterile tubes, centrifuged, and supernatants discarded. Pellets were resuspended in 1 mL ice-cold extraction buffer and lysed by sonication. After centrifugation (8000 × g, 10 min, 4°C), supernatants were collected with all extraction procedures performed on ice. Aliquots (200 μL) were transferred to 96-well plates at a density of 2×10⁴ cells/mL. LDH and CK-MB levels were quantified using commercial assay kits according to manufacturer protocols, with absorbance measured at 450 nm (Yang et al., 2017) .

**Determination of Compound 3b-1 Stability in PBS (HPLC Method)**

An appropriate amount of compound **3b-1** was accurately weighed, dissolved in phosphate-buffered saline (PBS, pH 7.4), and diluted to volume to prepare a stock solution at the target concentration (e.g., 100 µg/mL). Sample Grouping and Handling: The stock solution was aliquoted into multiple clean, sealed sample vials (HPLC injection vials). A sample designated as the 0-hour time point was analyzed immediately. Sampling at Time Points: Sample vials were retrieved at predetermined time points (0, 1, 5, and 12 hours). At the same time, stability testing was conducted on **3b-1** that was not dissolved in PBS but was stored in a dry state. HPLC Analysis: The sample solutions from each time point were immediately subjected to HPLC analysis. Chromatographic Conditions: C18 column (150 mm × 4.6 mm, 5 µm); Mobile phase: methanol/water = 80:20 (v/v); Flow rate: 1.0 mL/min; Detection wavelength: 254 nm; Column temperature: 30°C; Injection volume: 10 µL. Data Processing: The peak area of compound **3b-1** was recorded. The peak area retention rate (%) for each time point was calculated relative to the 0-hour peak area (set as 100%). The decreasing trend of the retention rate over time was observed to assess the chemical stability of compound **3b-1** in PBS.


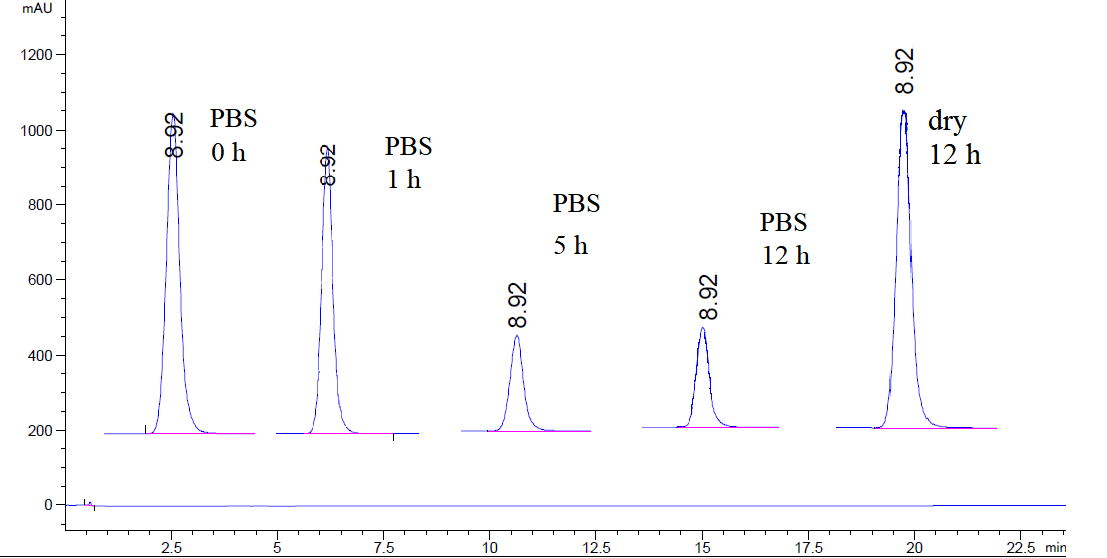


**Figure S1**. Stability testing of compound **3b-1**.

**Spectral data**


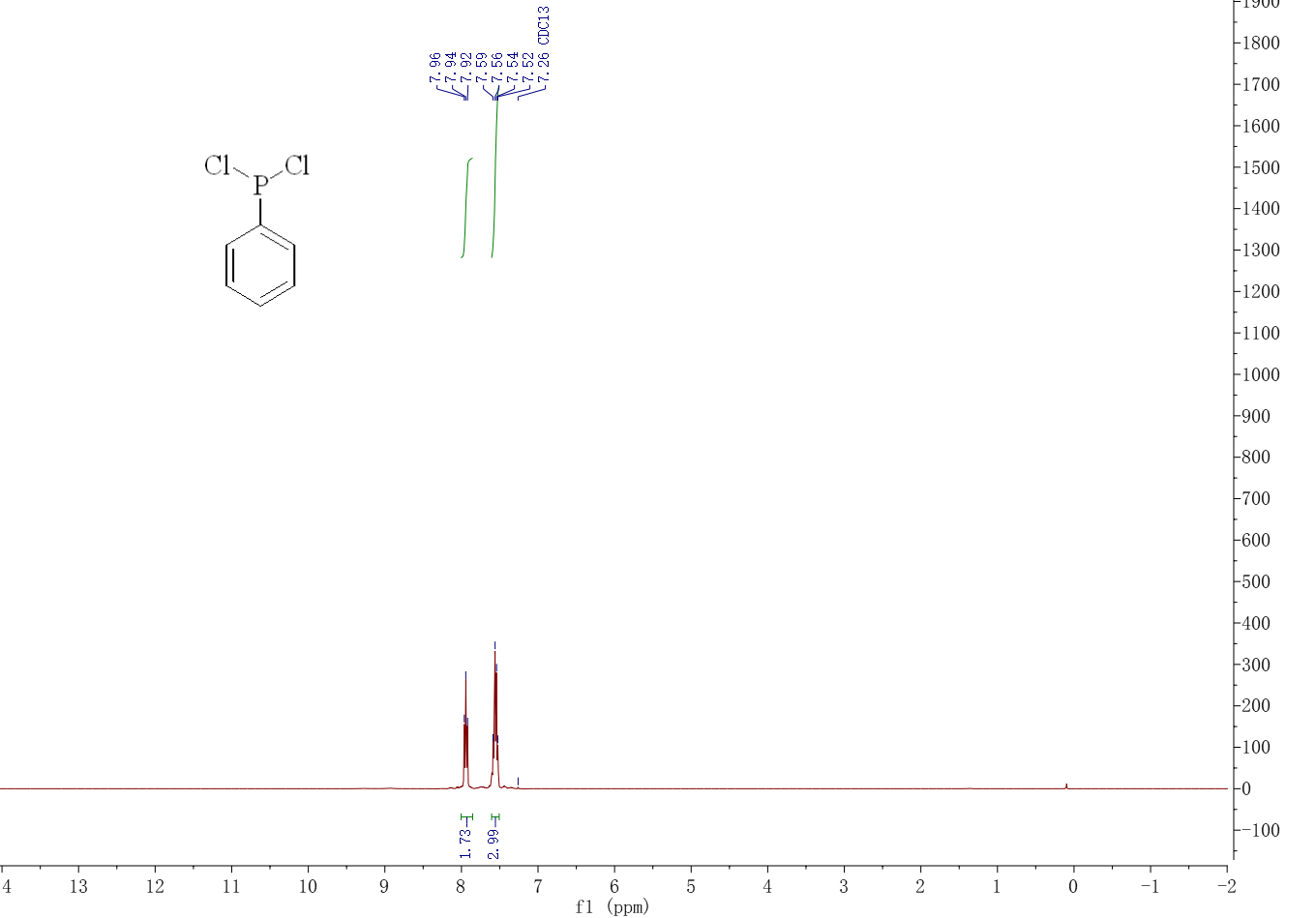


Fig 1. *^1^H NMR of* **1** (400 MHz, CDCl_3_)


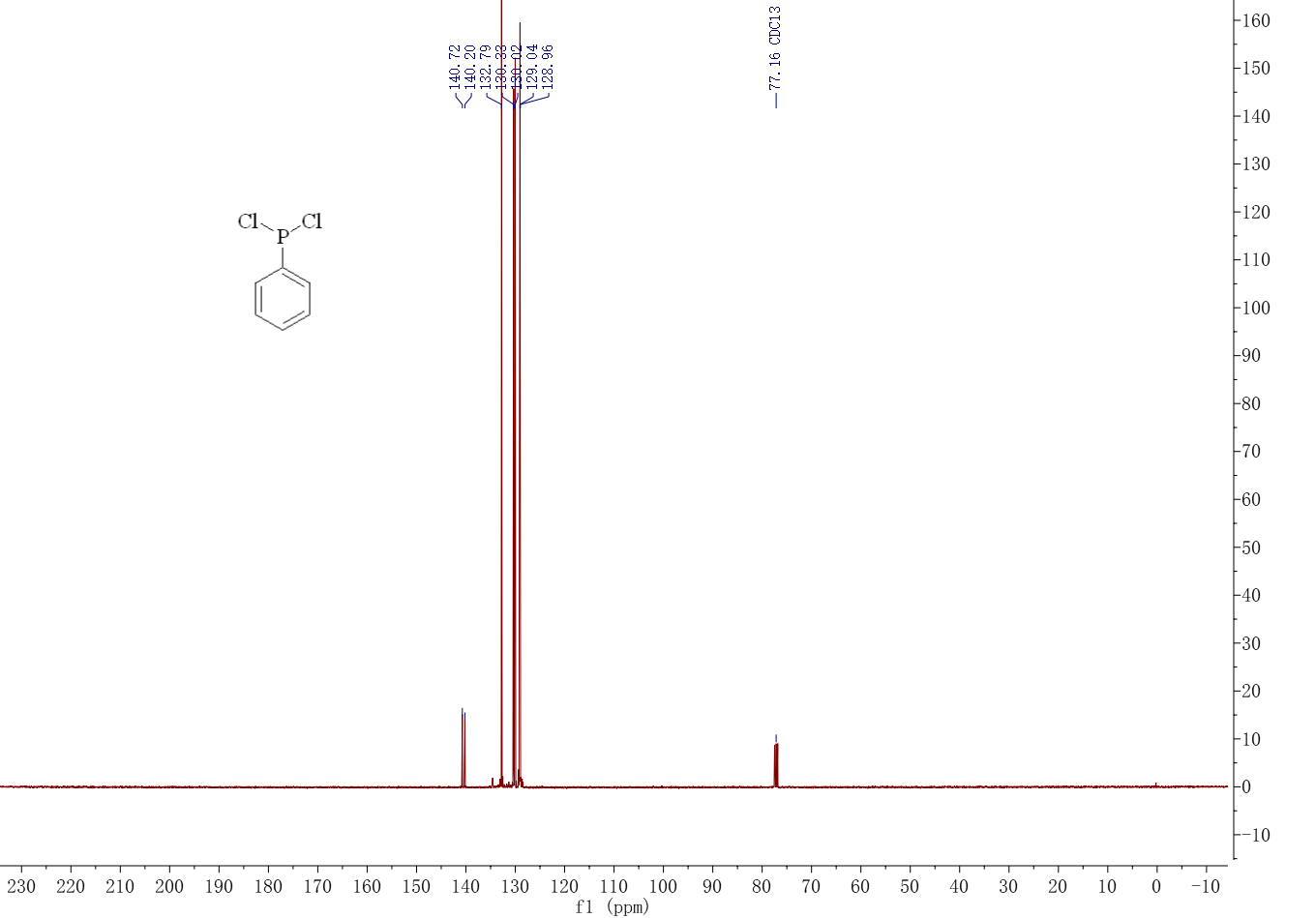


Fig 2. *^13^C NMR of* **1** (101 MHz, CDCl_3_)


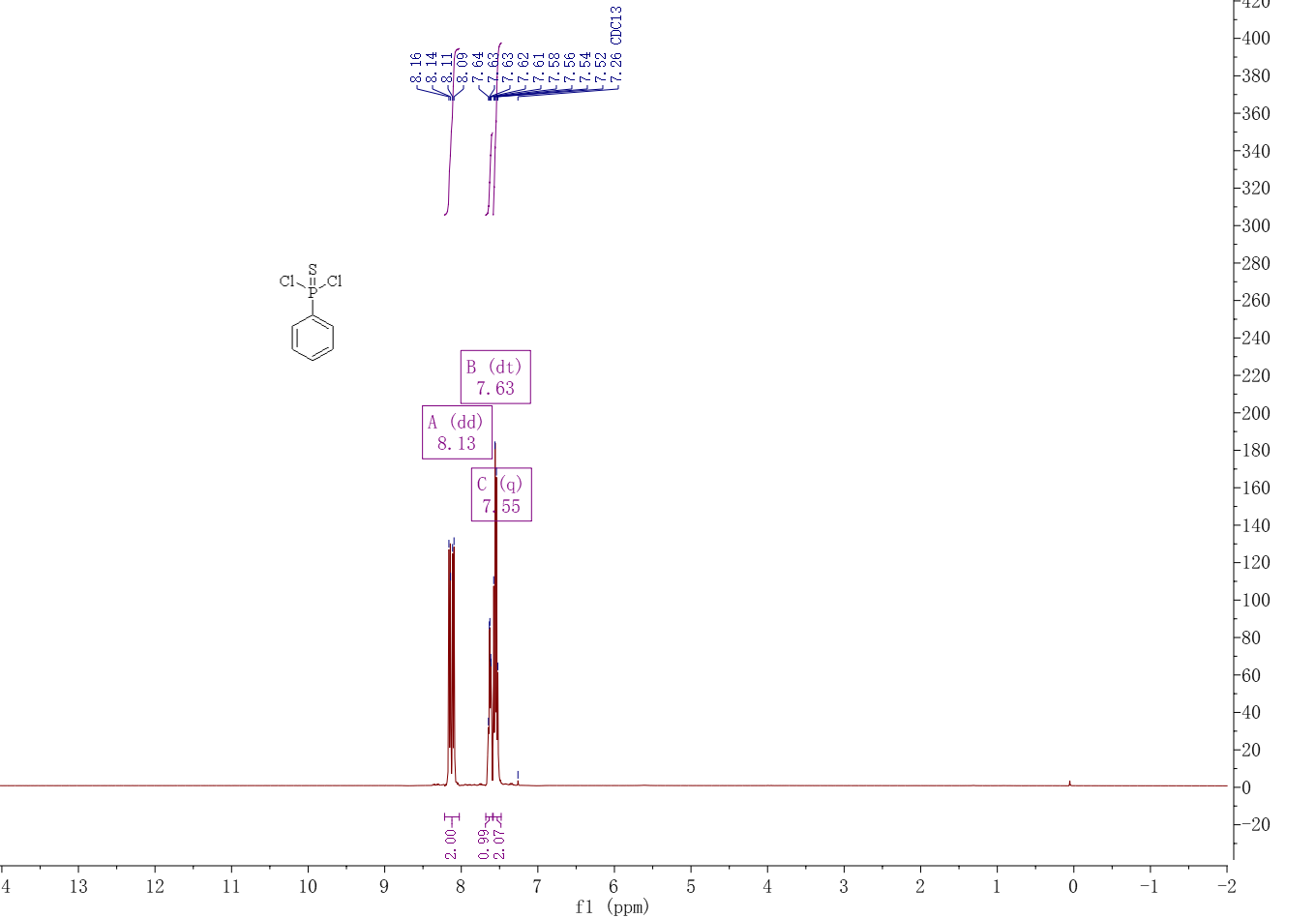


Fig 3. *^1^H NMR of* **2** (400 MHz, CDCl_3_)


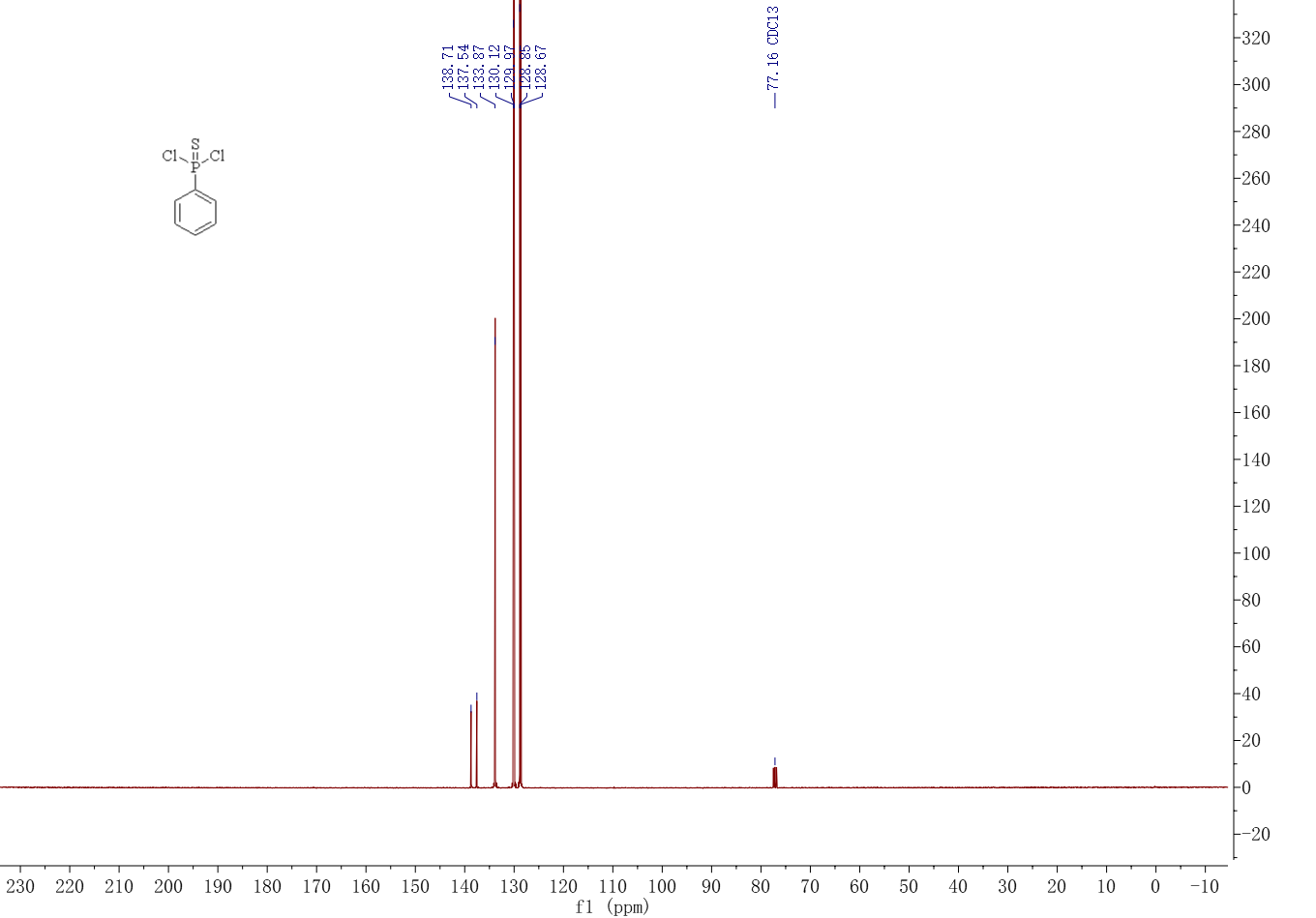


Fig 4. *^13^C NMR of* **2** (101 MHz, CDCl_3_)


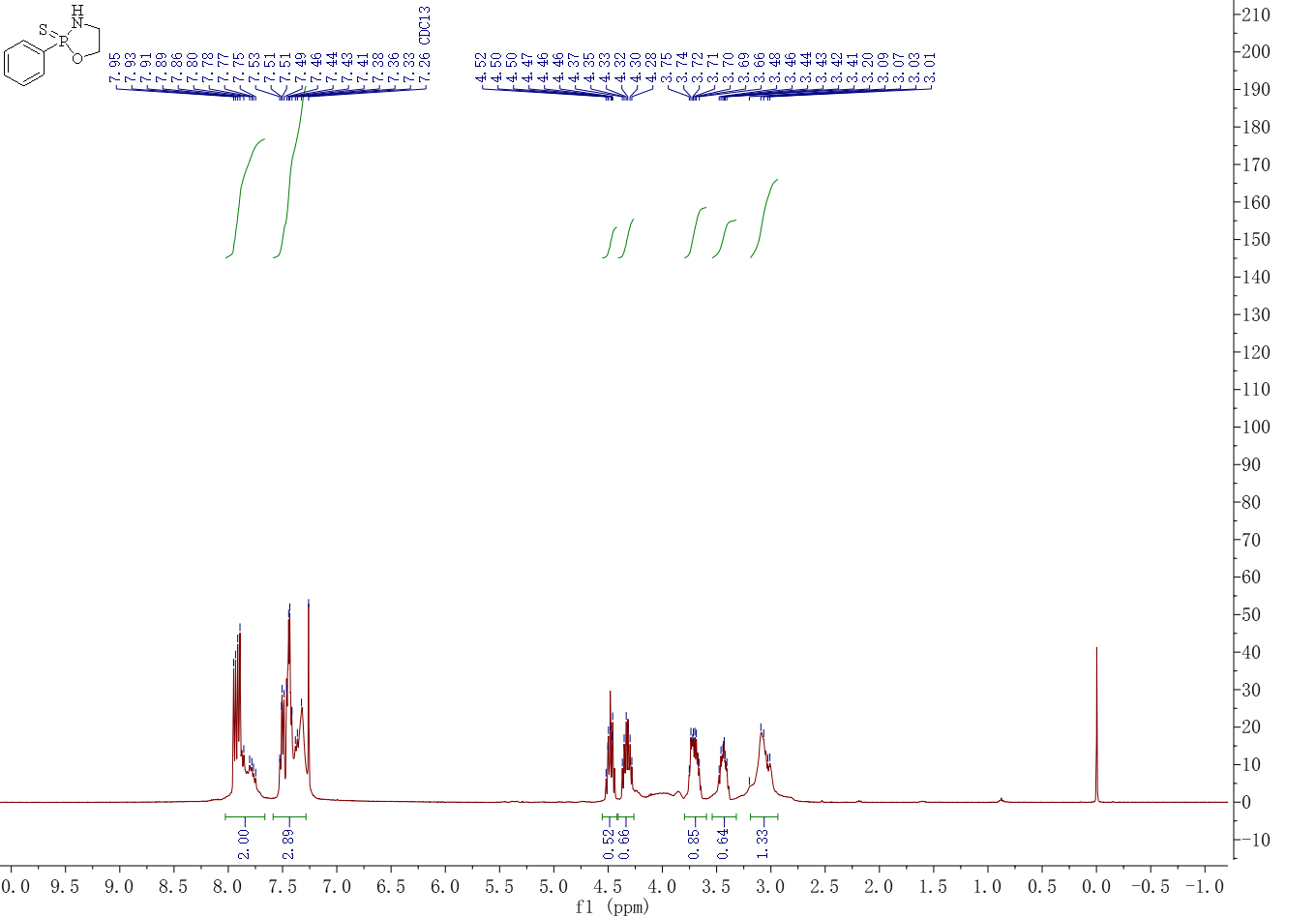


Fig 5. *^1^H NMR of* **3a-1** (400 MHz, CDCl_3_)


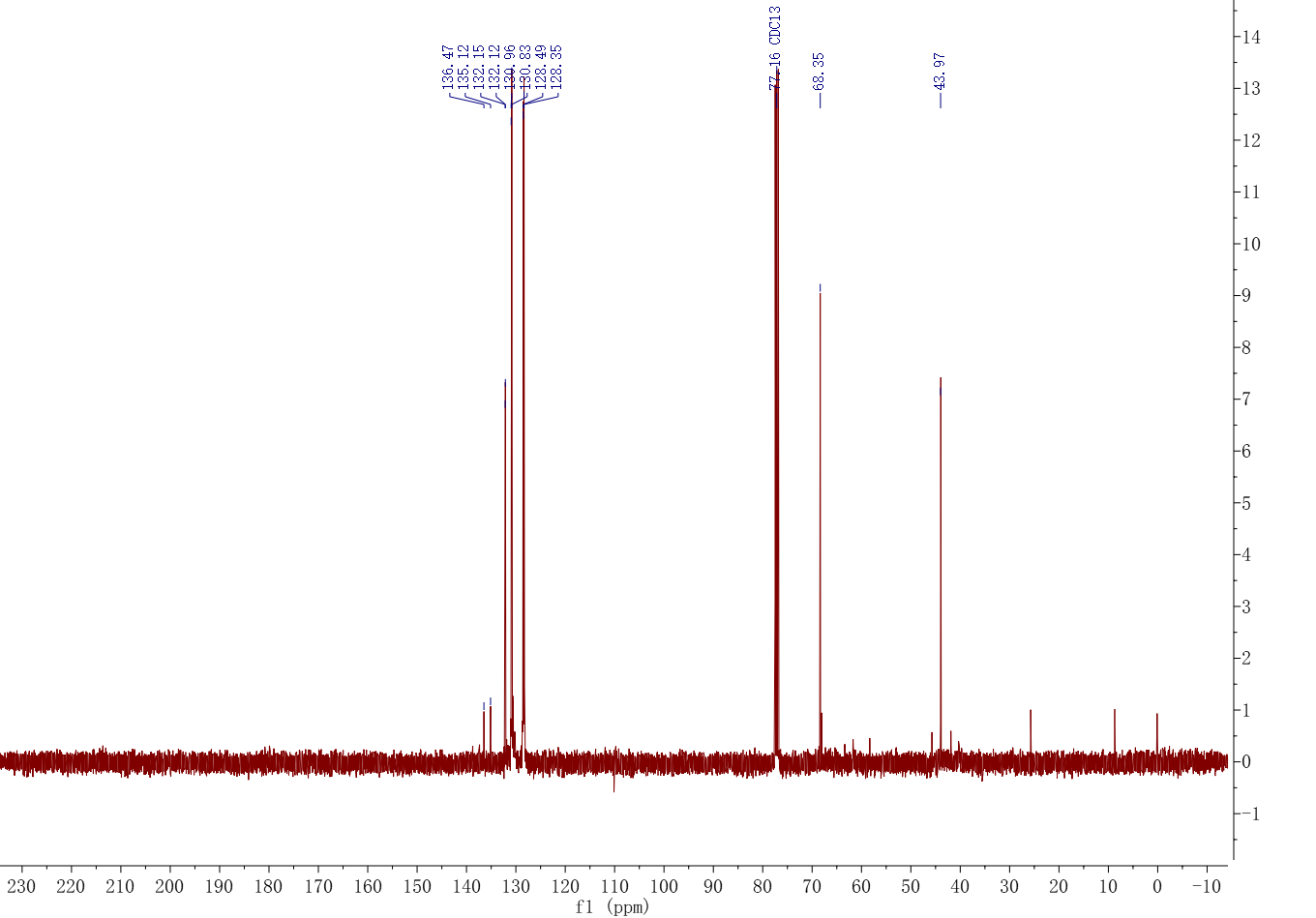


Fig 6. *^13^C NMR of* **3a-1** (101 MHz, CDCl_3_)


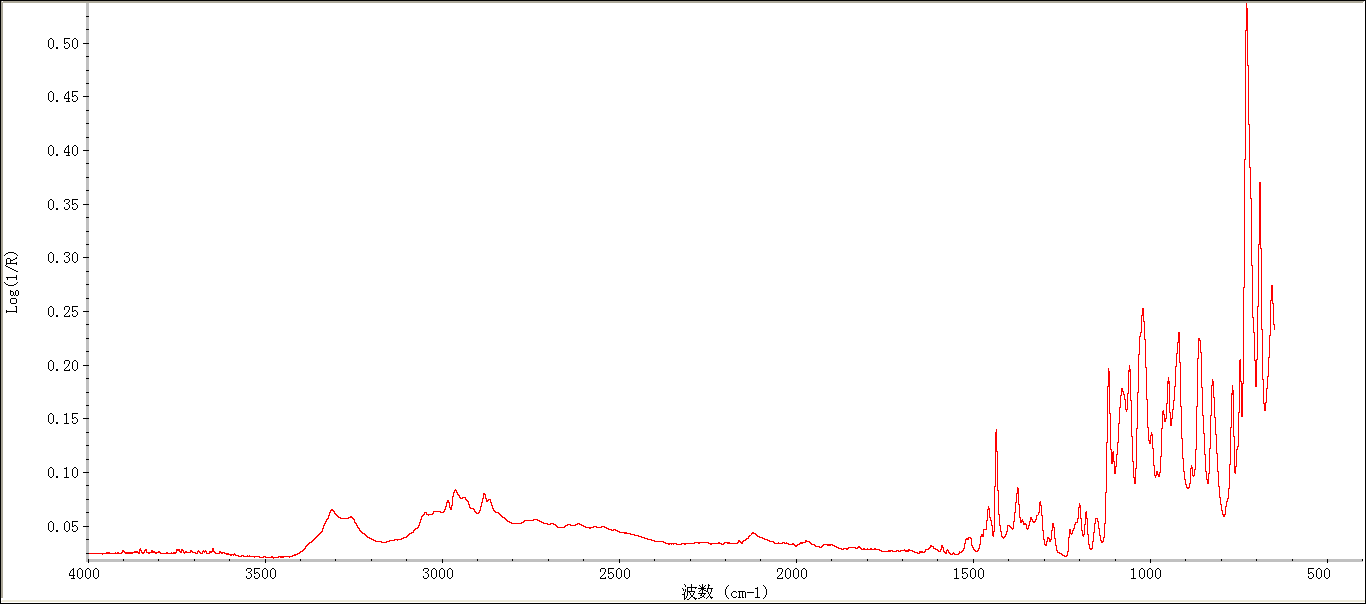


Fig 7. The FTIR spectrum of **3a-1**


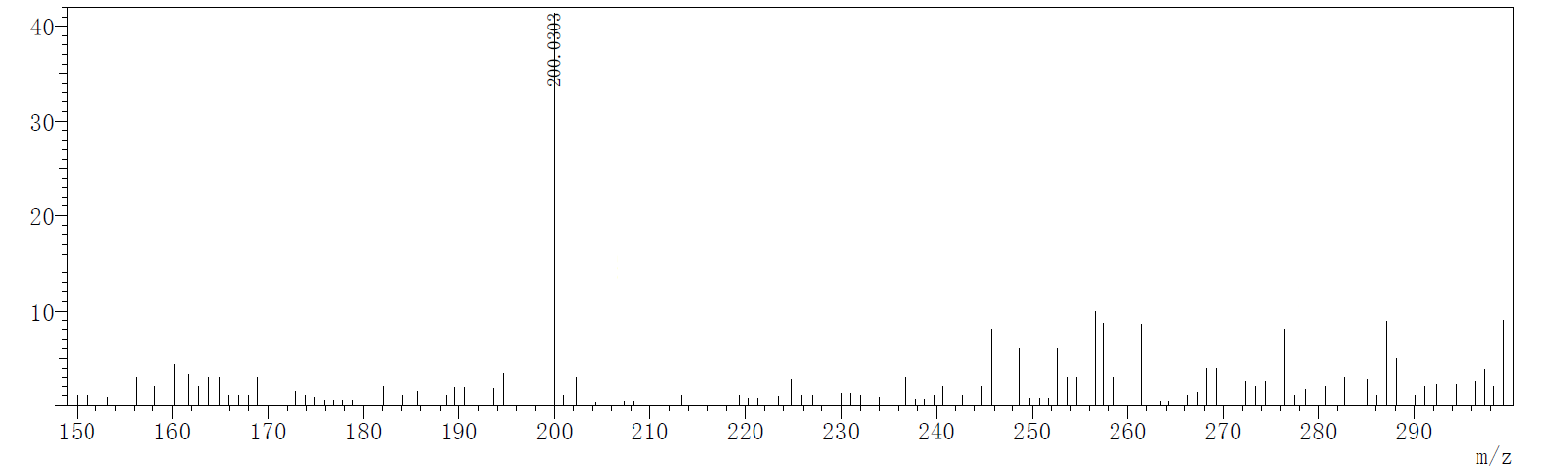


Fig 8. *Mass spectrum of compound* **3a-1**


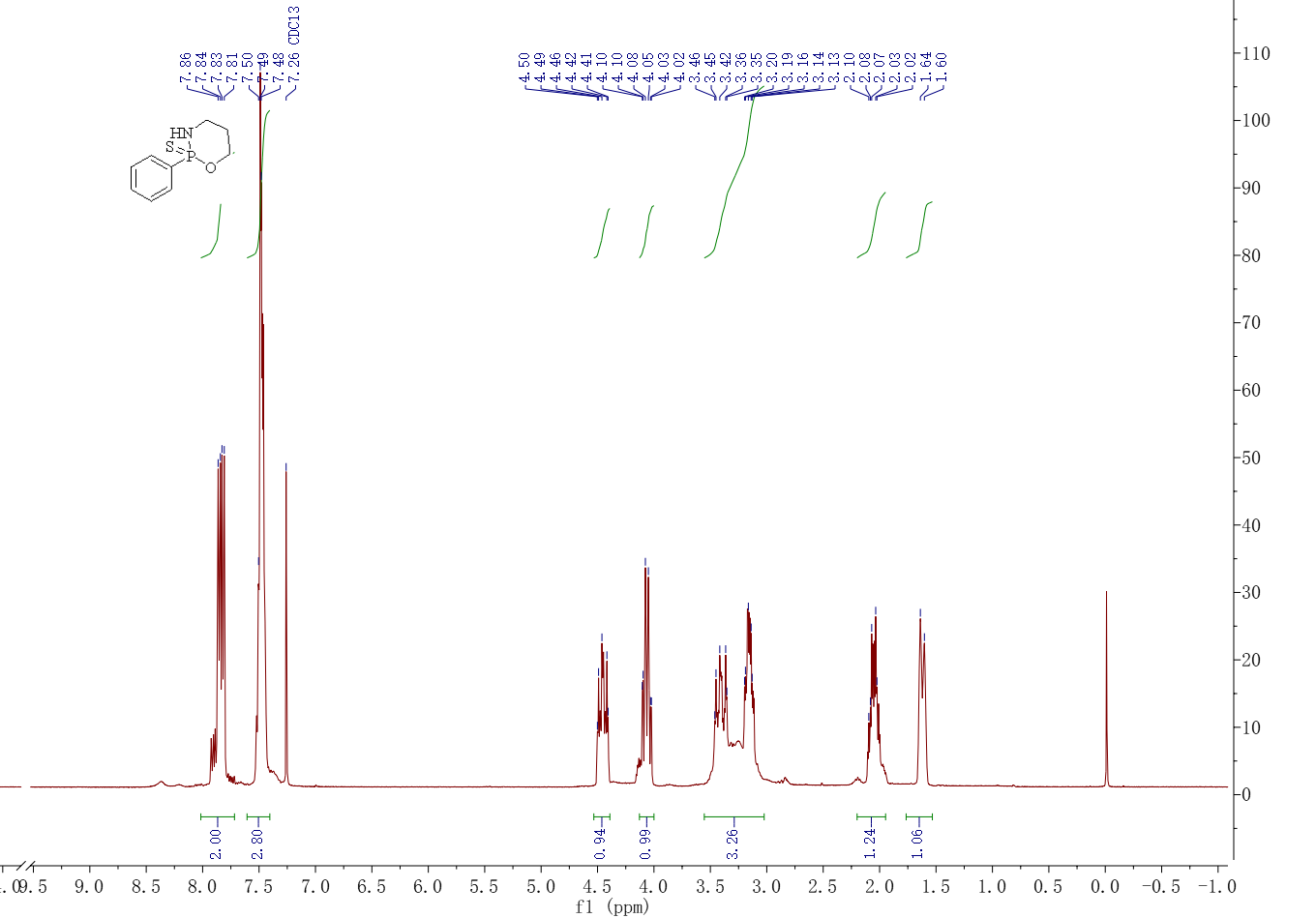


Fig 9. *^1^H NMR of* **3a-2** (400 MHz, CDCl_3_)


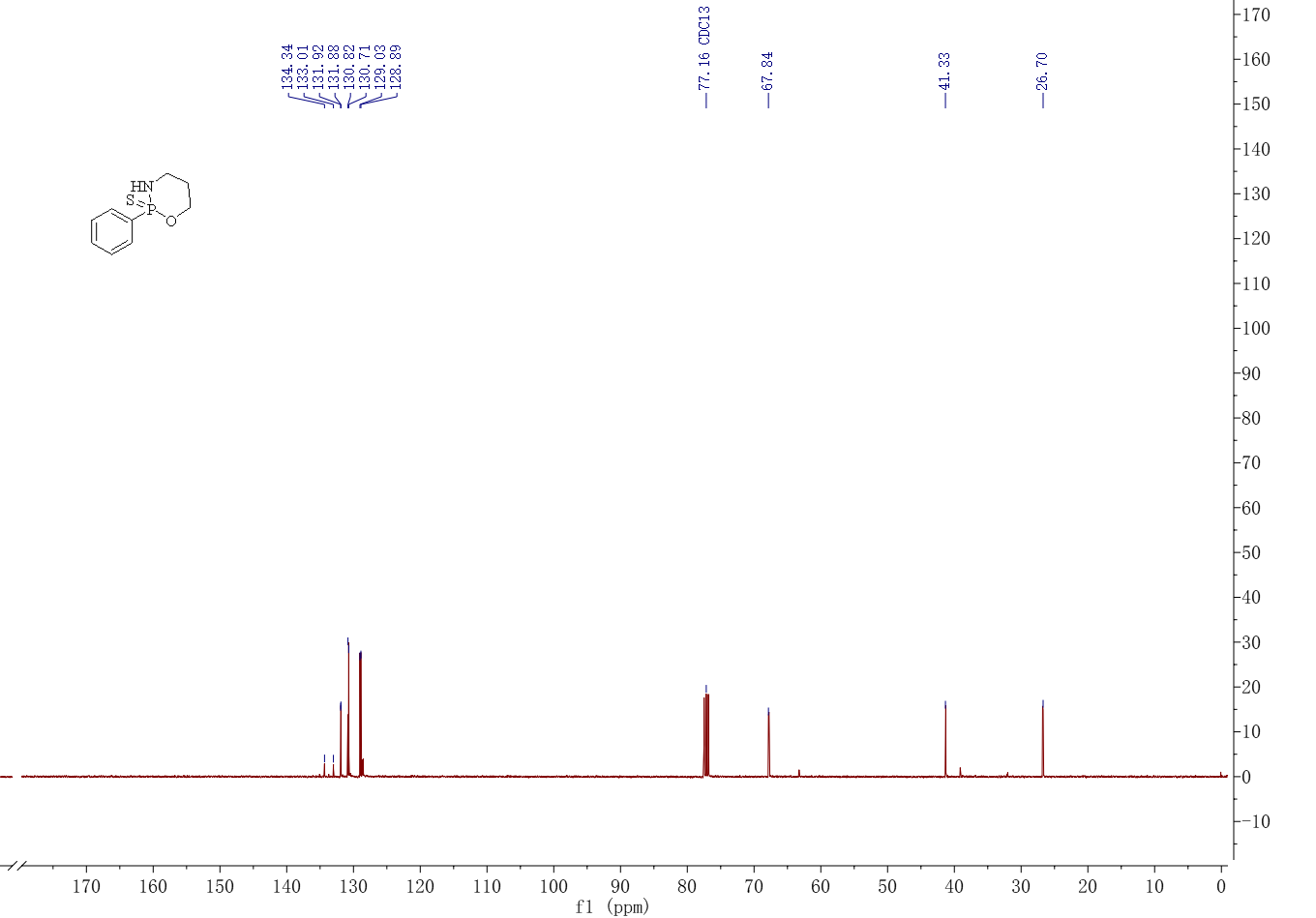


Fig 10. *^13^C NMR of* **3a-2** (101 MHz, CDCl_3_)


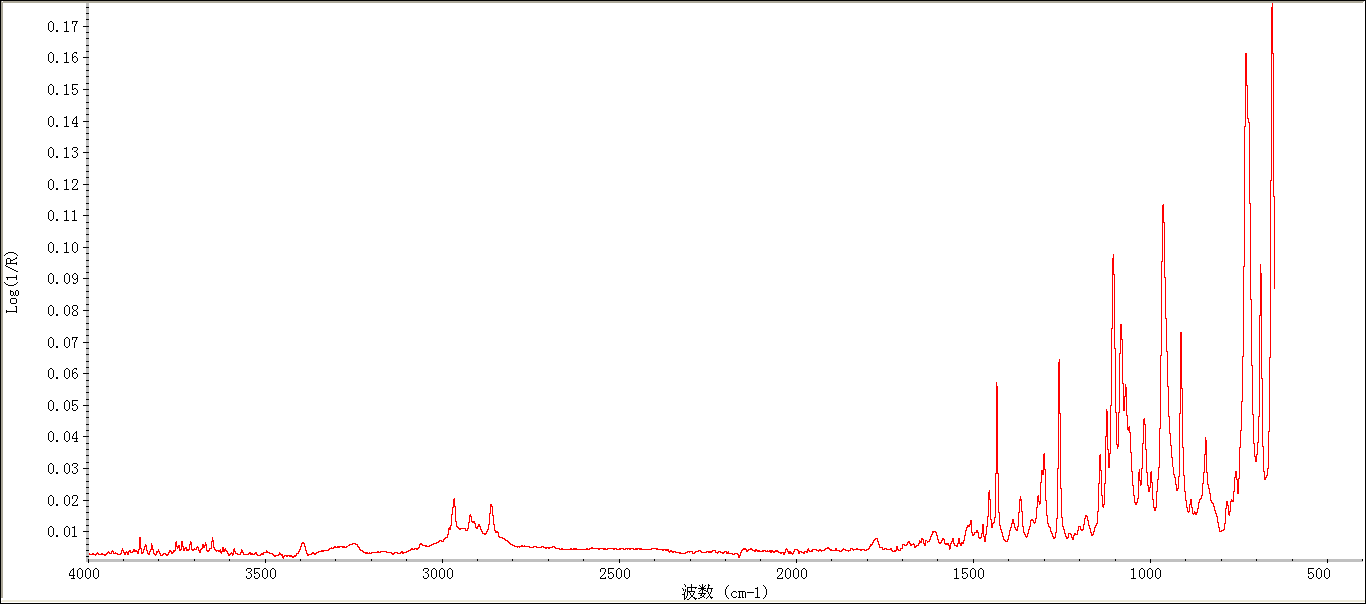


Fig 11. The FTIR spectrum of **3a-2**


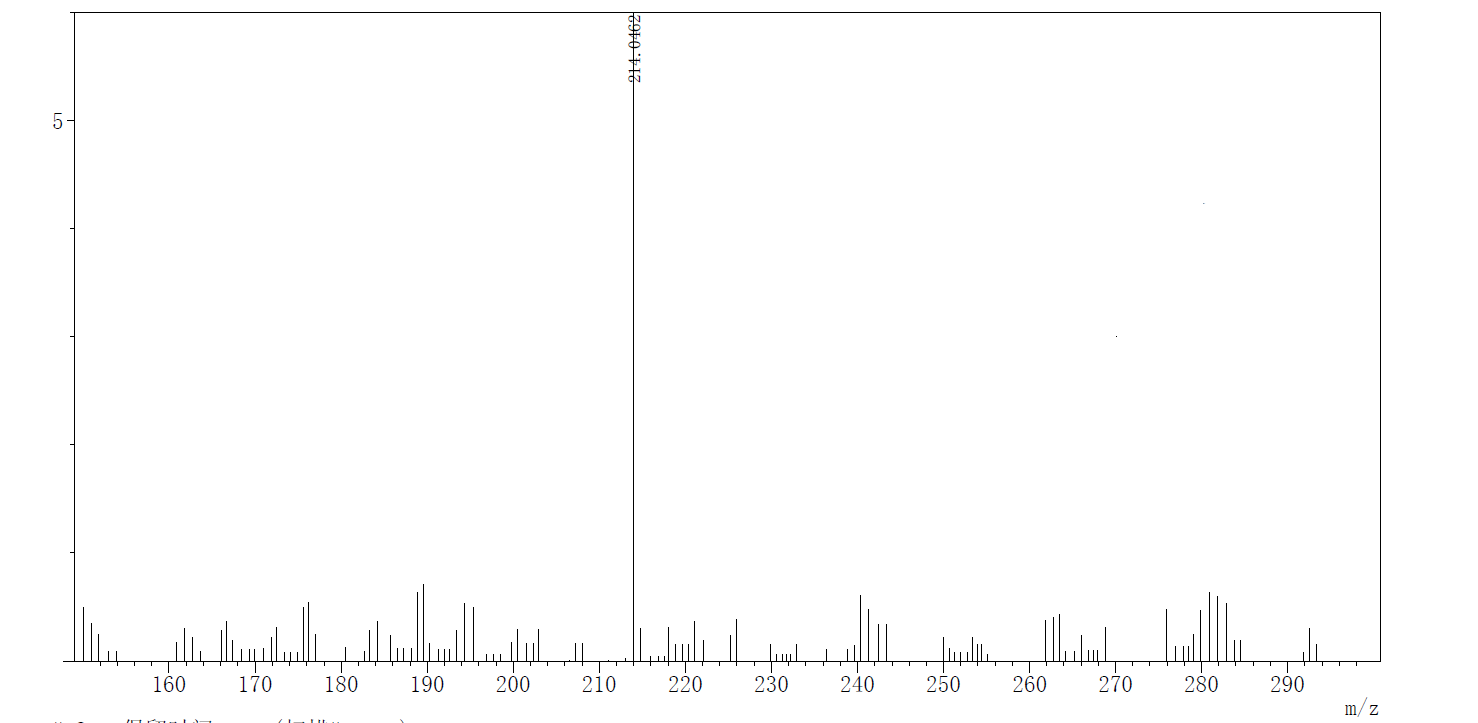


Fig 12. *Mass spectrum of compound* **3a-2**


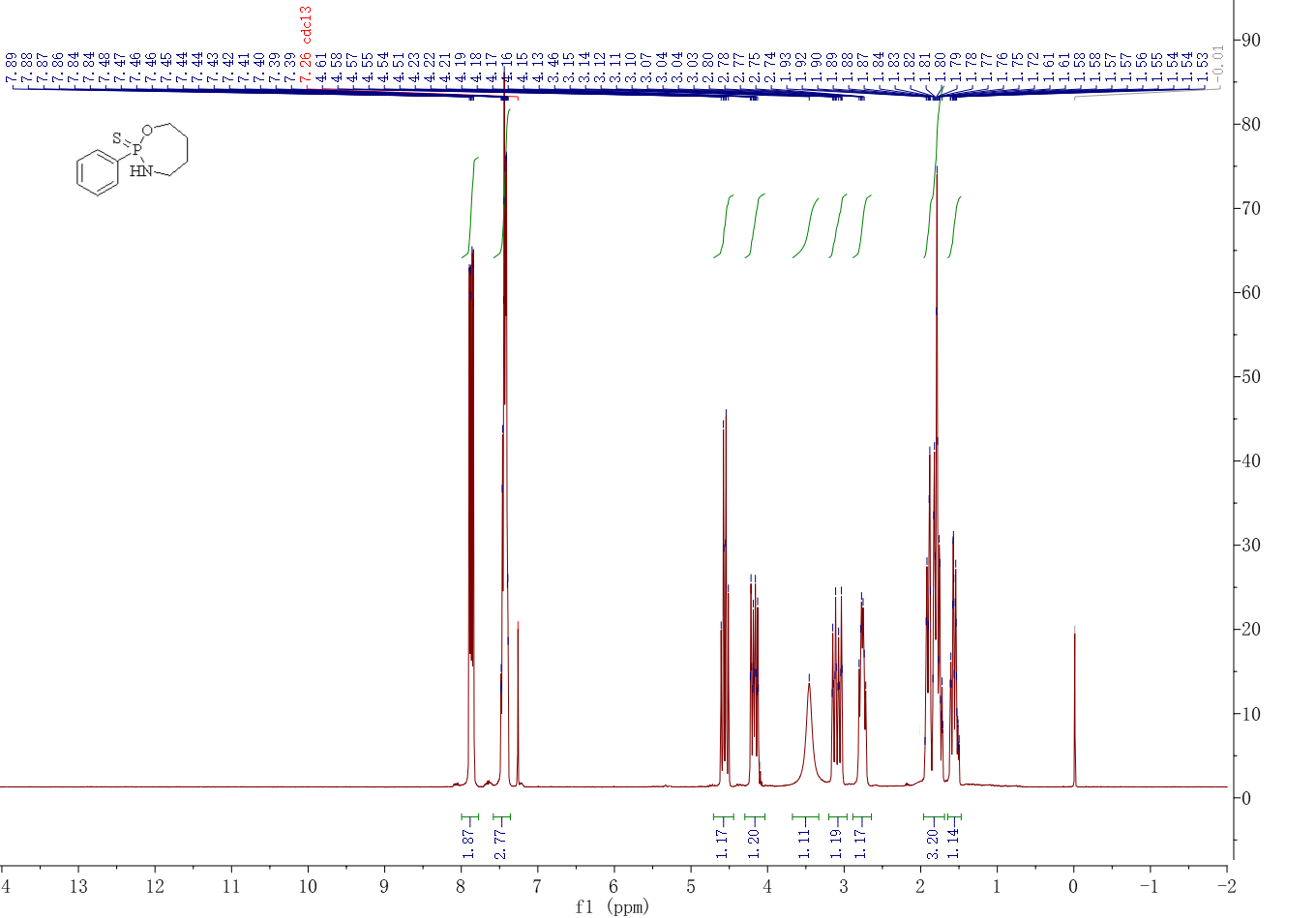


Fig 13. *^1^H NMR of* **3a-3** (400 MHz, CDCl_3_)


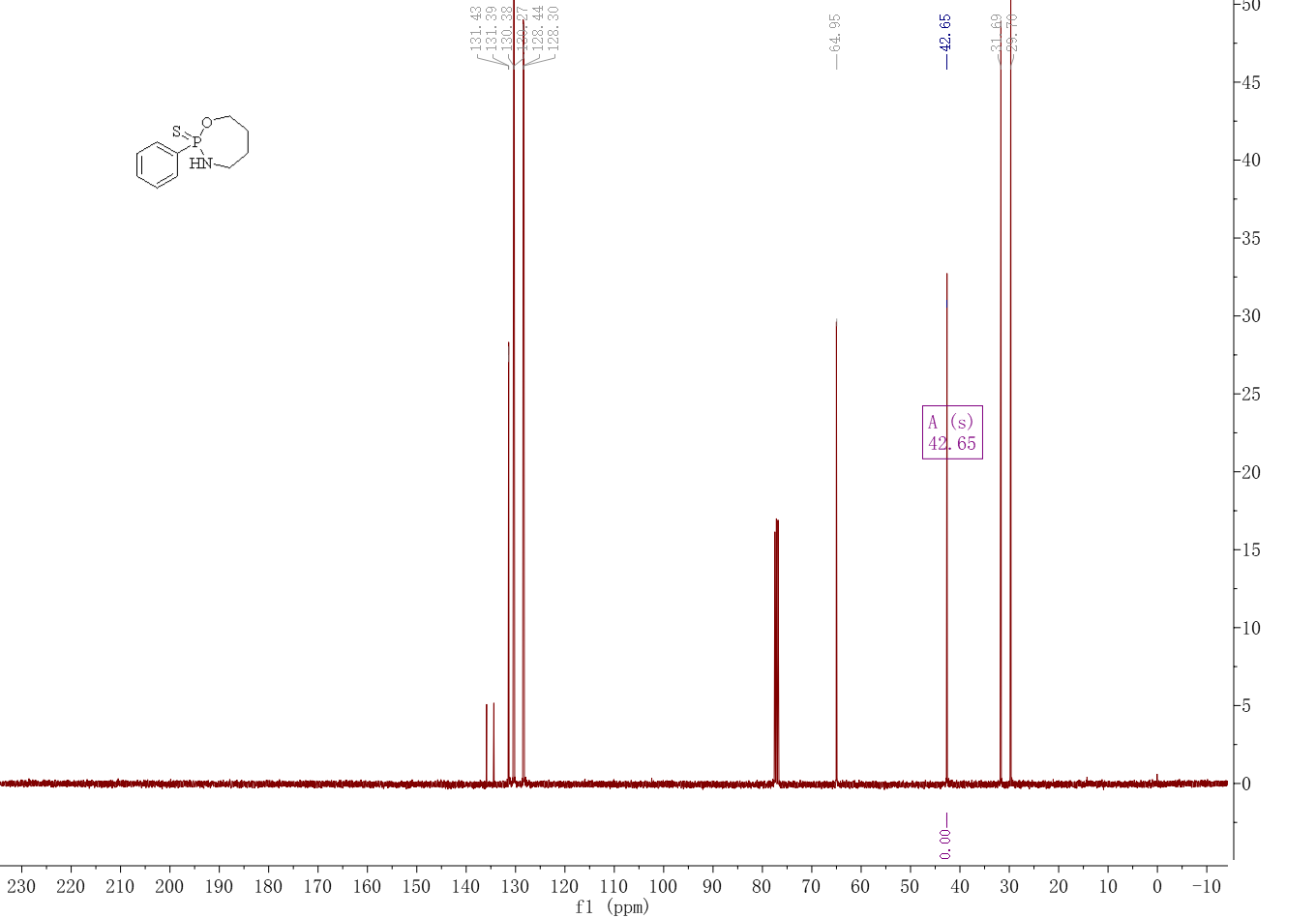


Fig 14. *^13^C NMR of* **3a-3** (101 MHz, CDCl_3_)


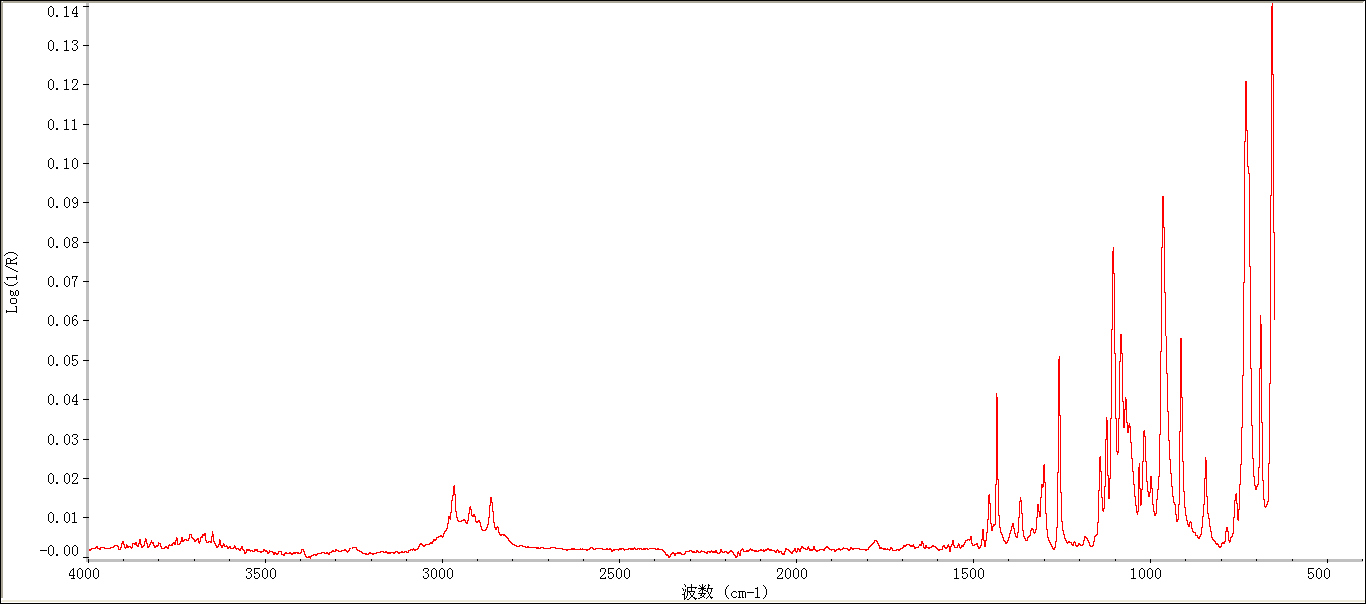


Fig 5. The FTIR spectrum of **3a-3**


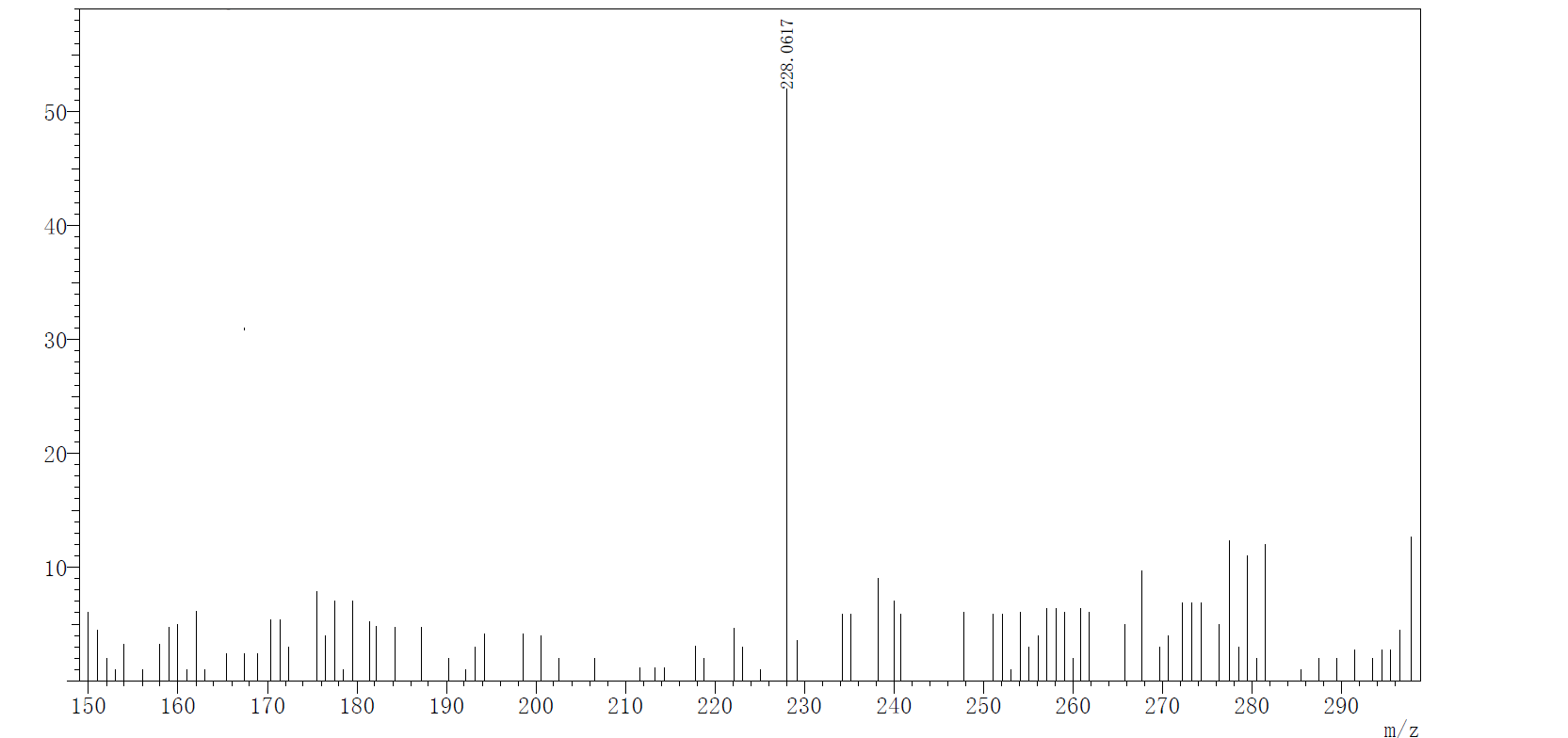


Fig 16. *Mass spectrum of compound* **3a-3**


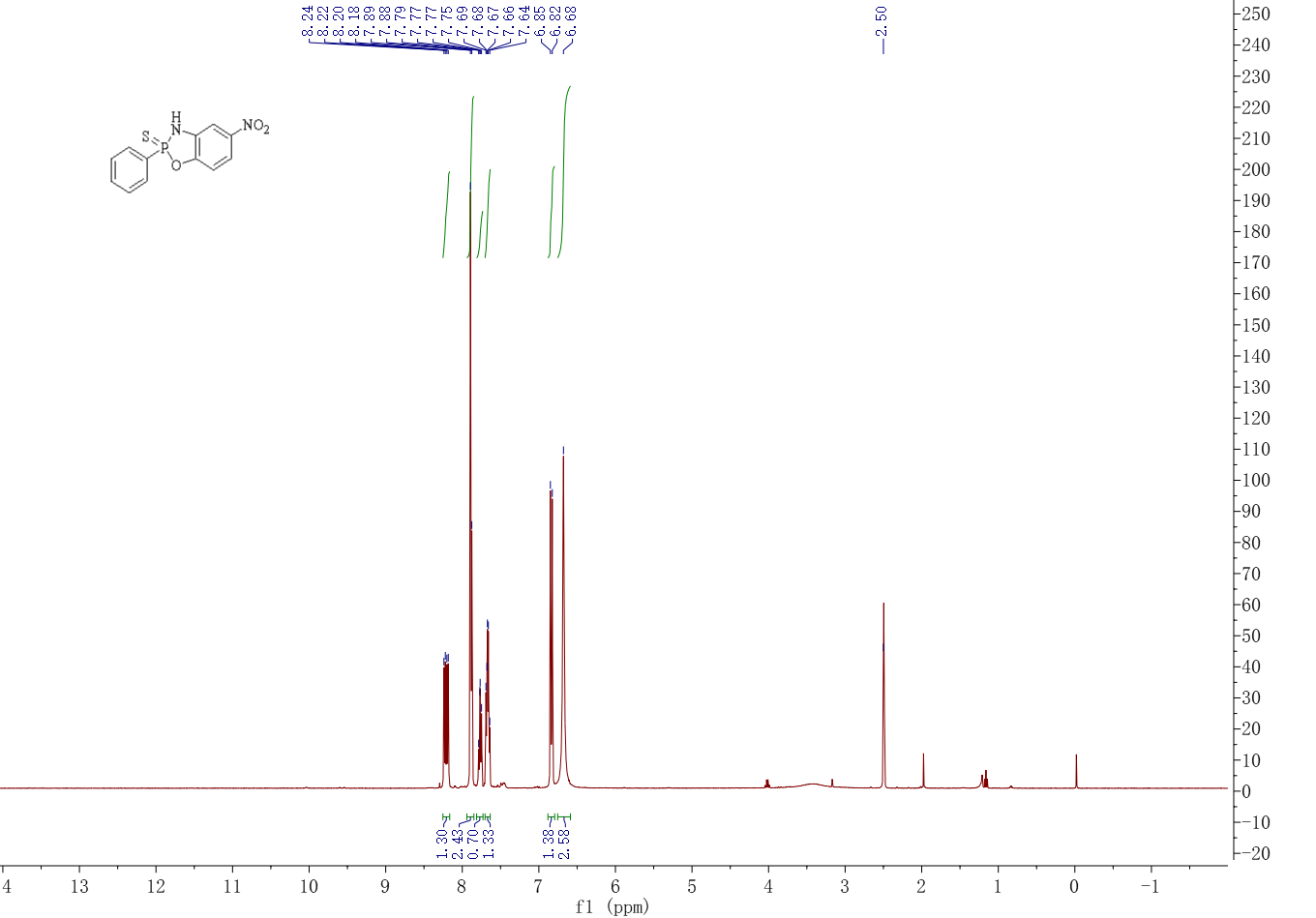


Fig 17. *^1^H NMR of* **3b-1** (400 MHz, DMSO)


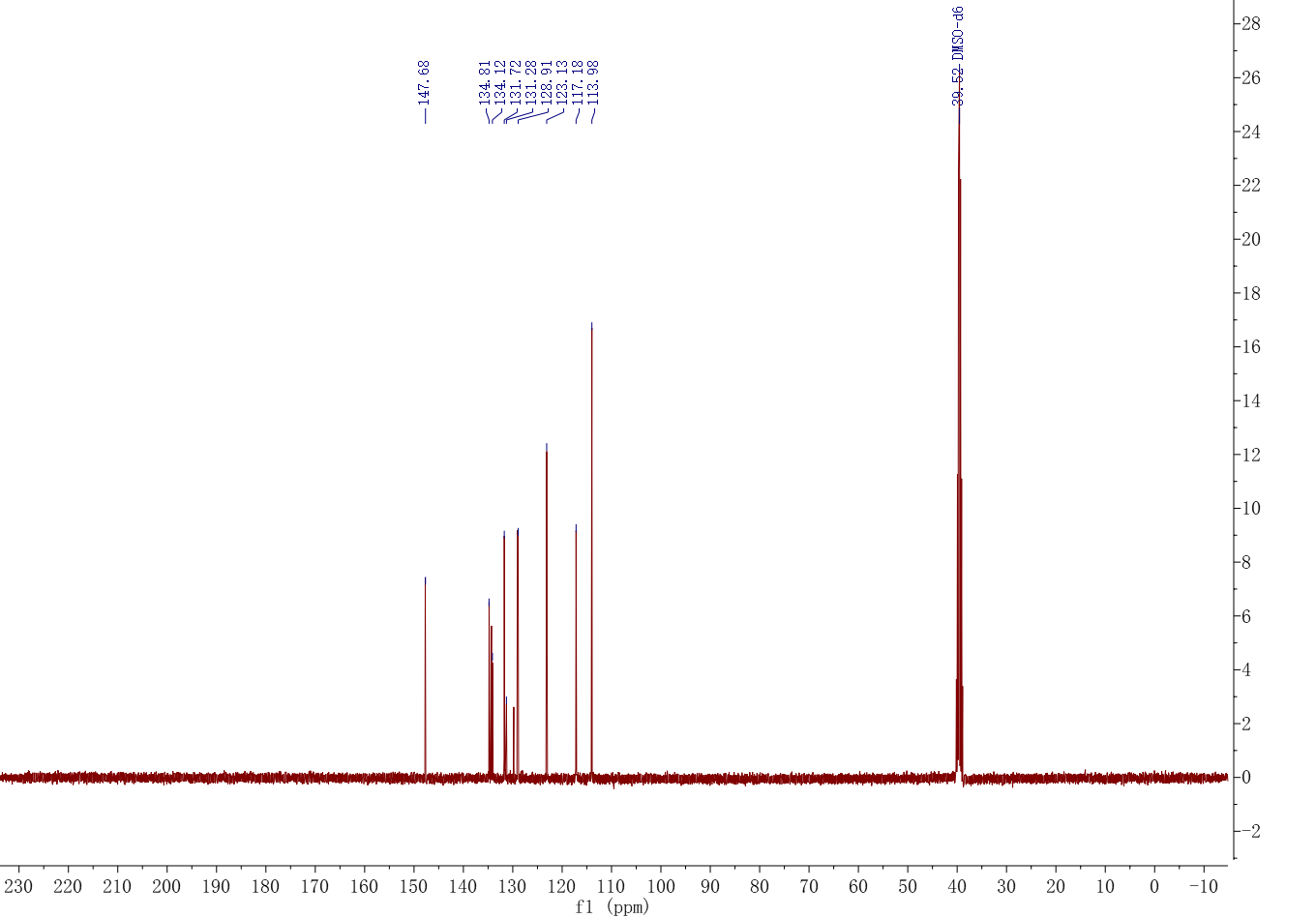


Fig 18. *^13^C NMR of* **3b-1** (101 MHz, DMSO)


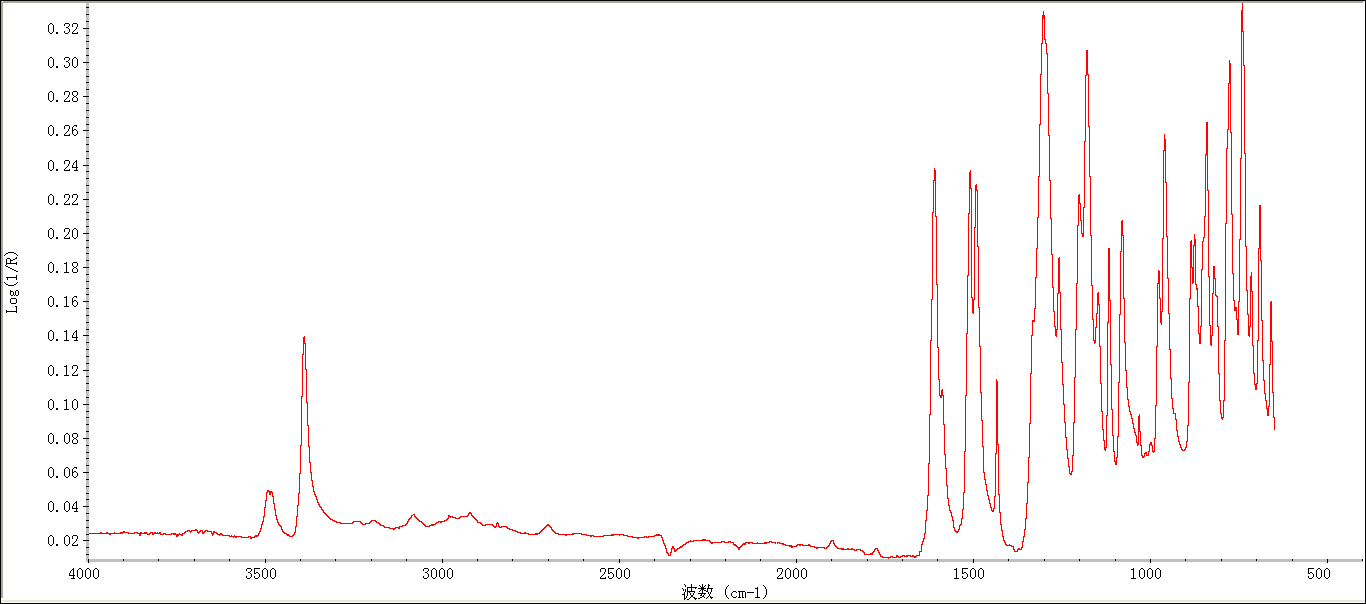


Fig 19. The FTIR spectrum of **3b-1**


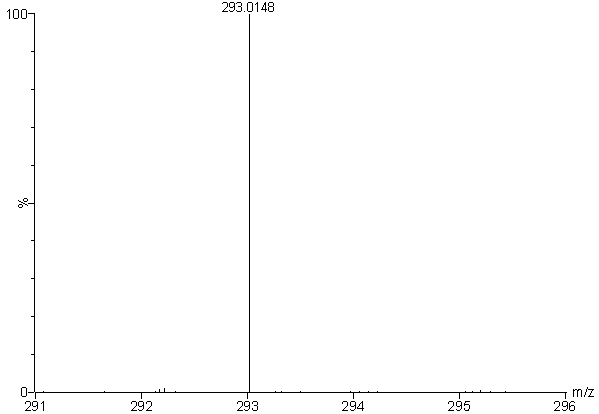


Fig 20. *Mass spectrum of compound* **3b-1**


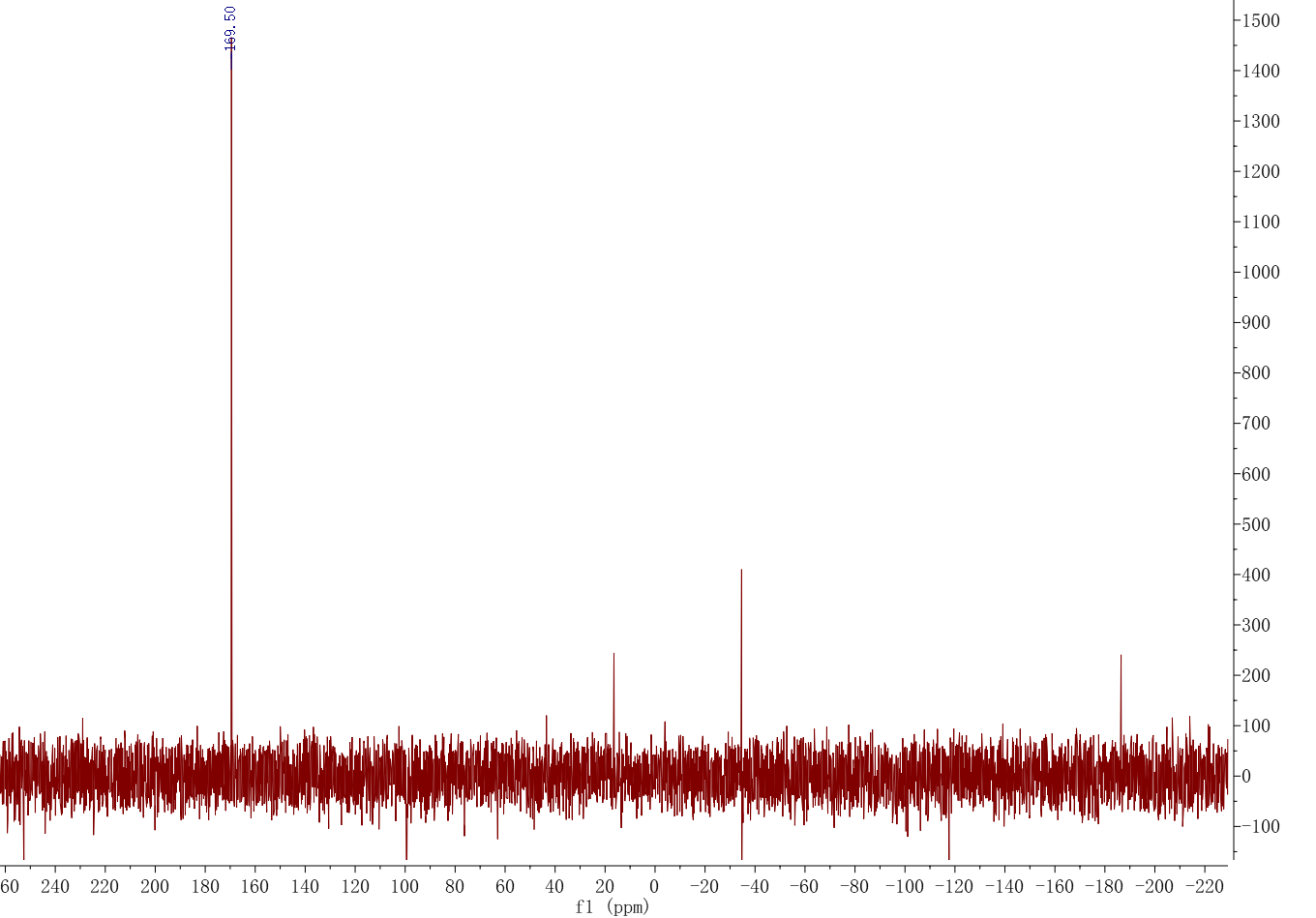


Fig 21. *^31^P NMR of* **3b-1** (162 MHz, DMSO)


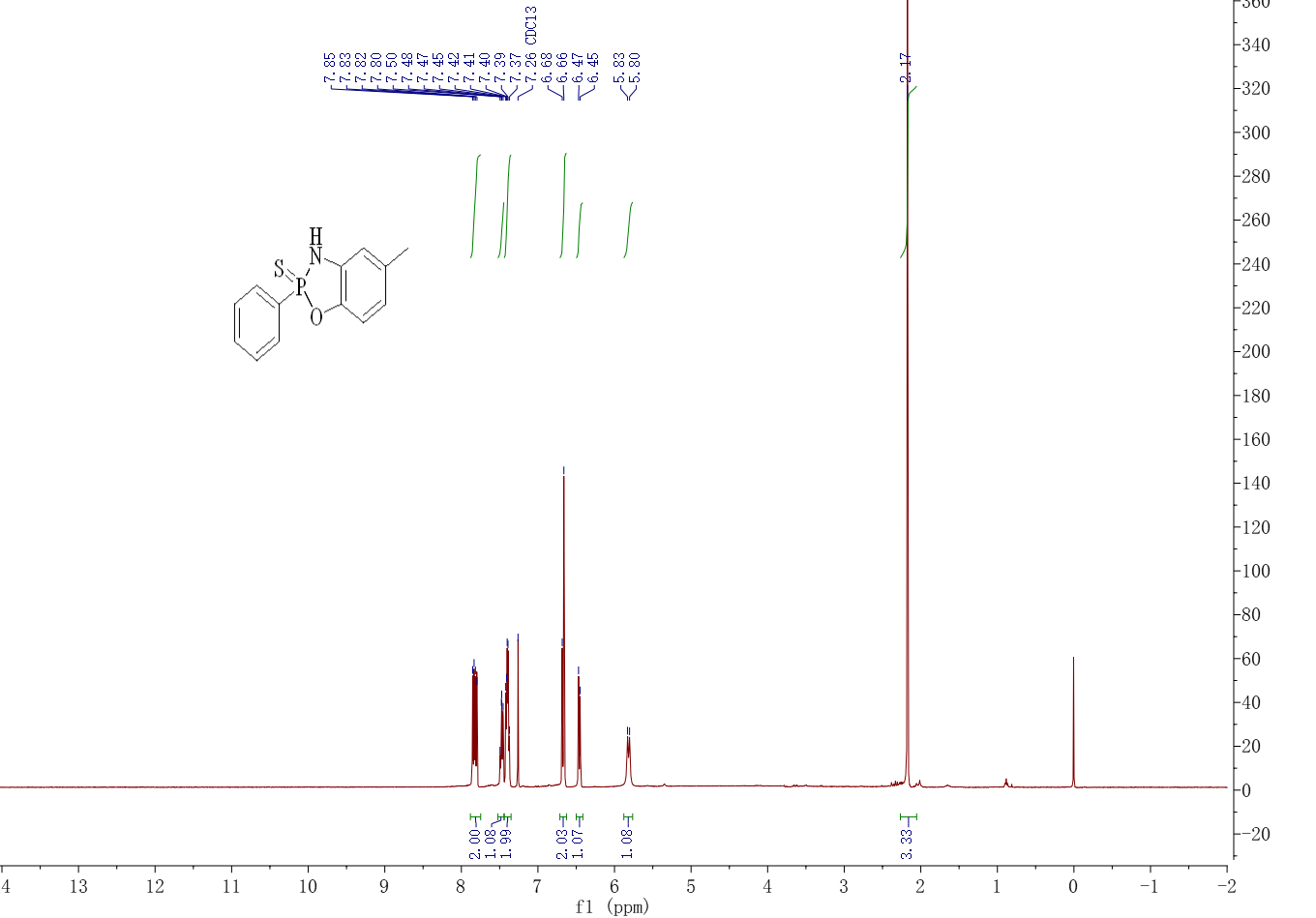


Fig 22. *^1^H NMR of* **3b-2** (400 MHz, CDCl_3_)


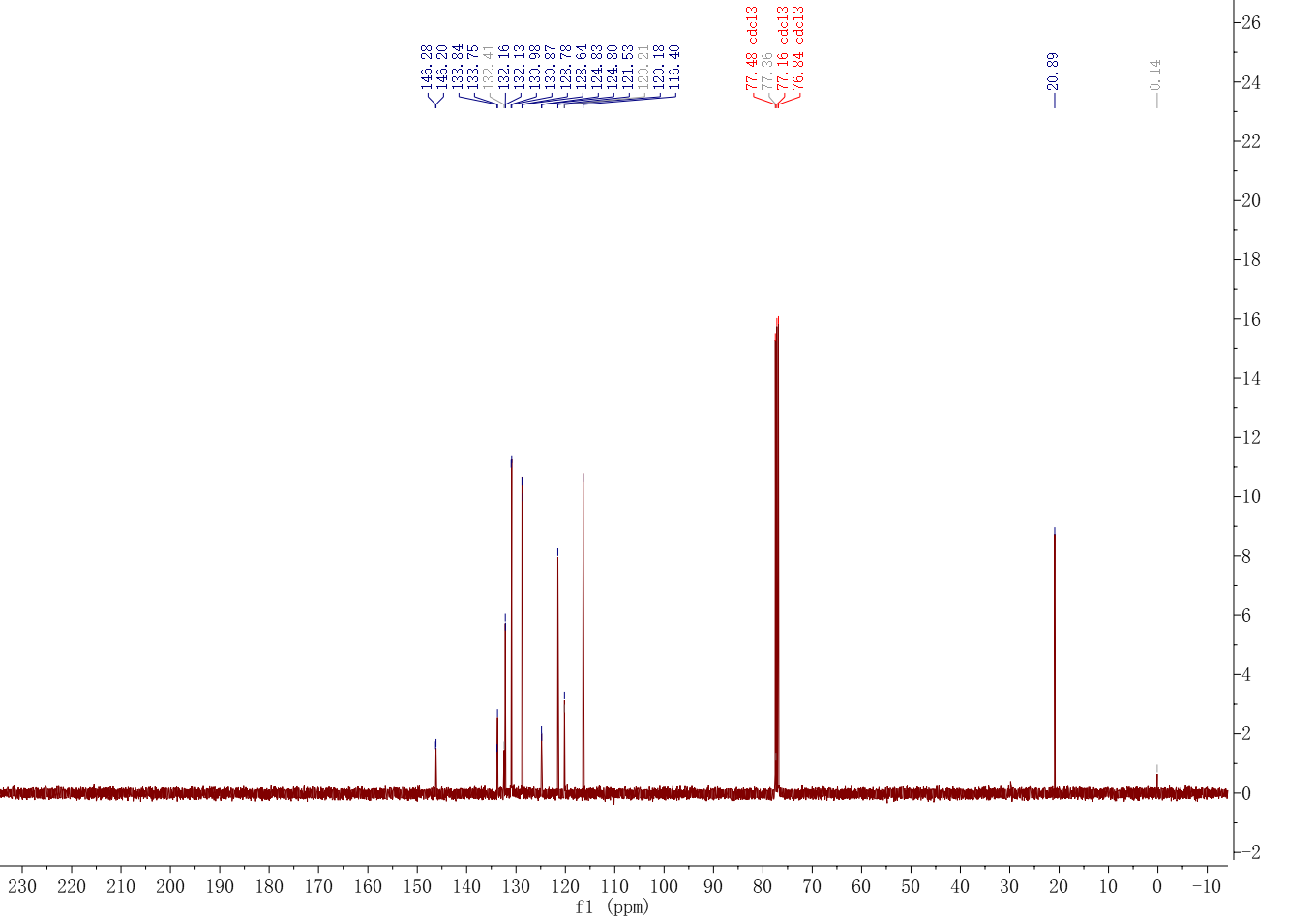


Fig 23. *^13^C NMR of* **3b-2** (100 MHz, CDCl_3_)


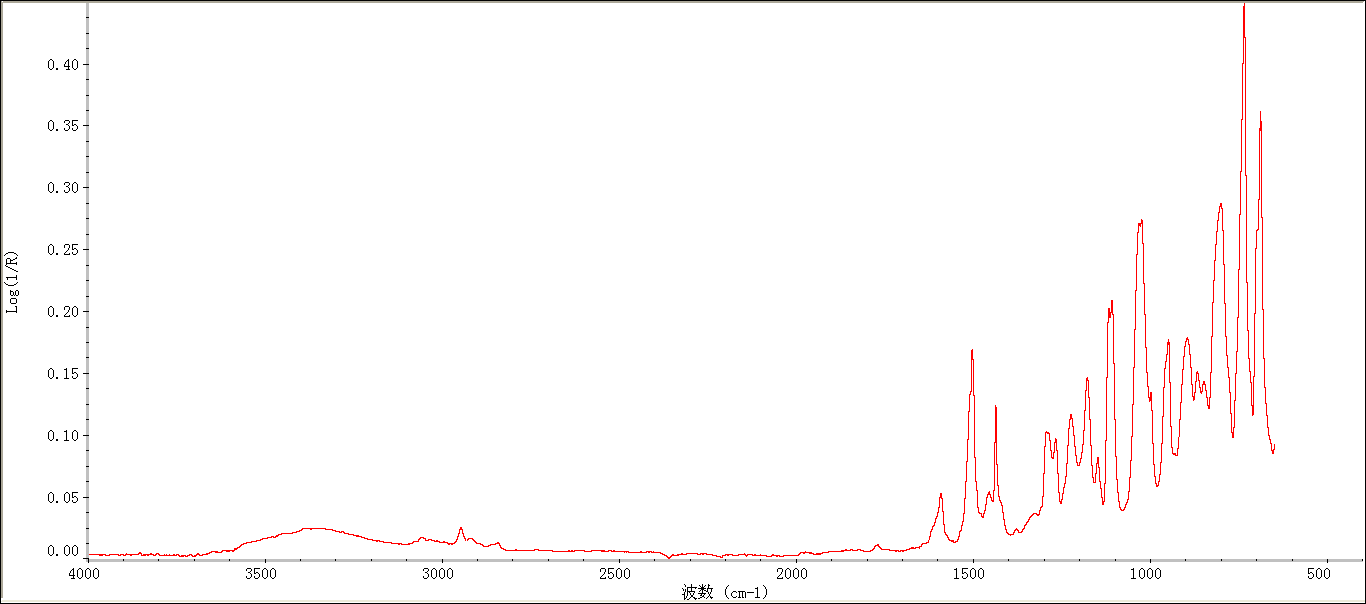


Fig 24. The FTIR spectrum of **3b-2**


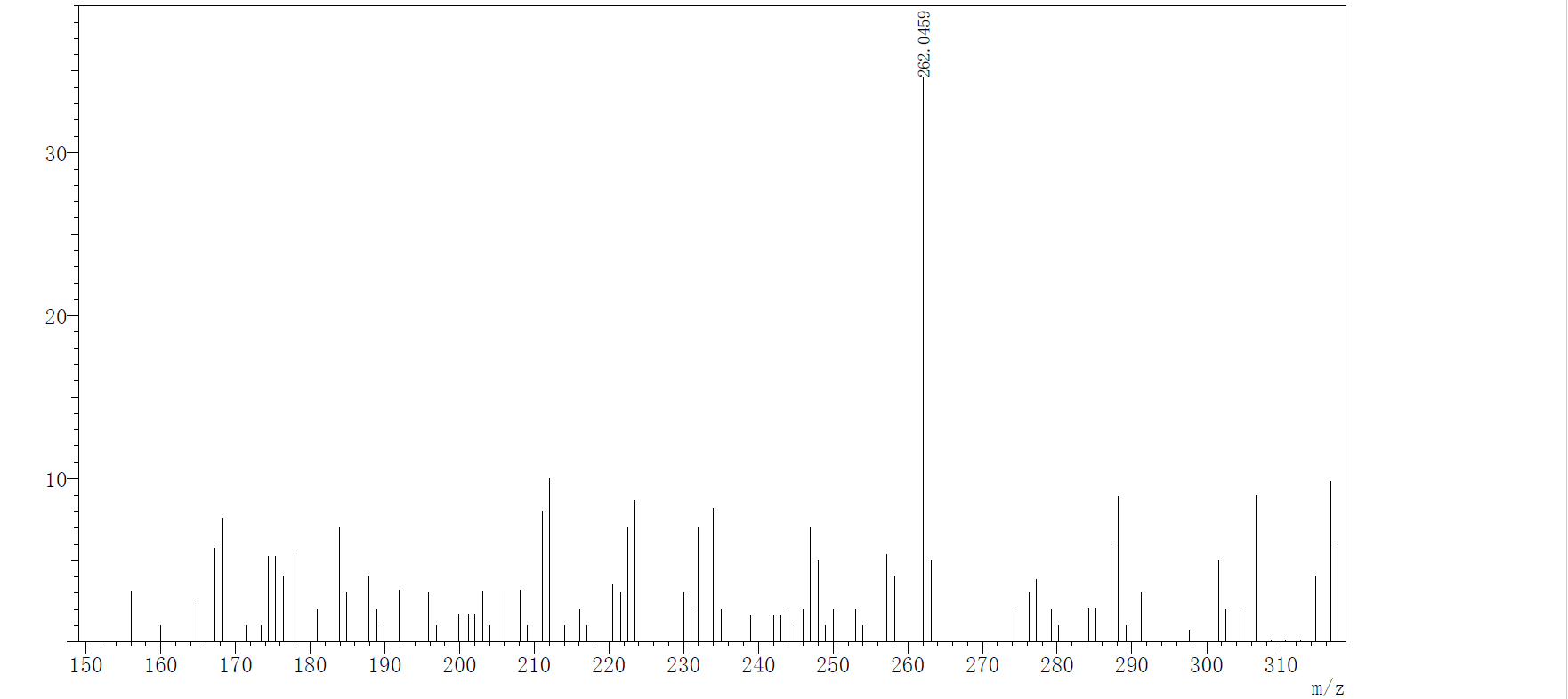


Fig 25. *Mass spectrum of compound* **3b-2**


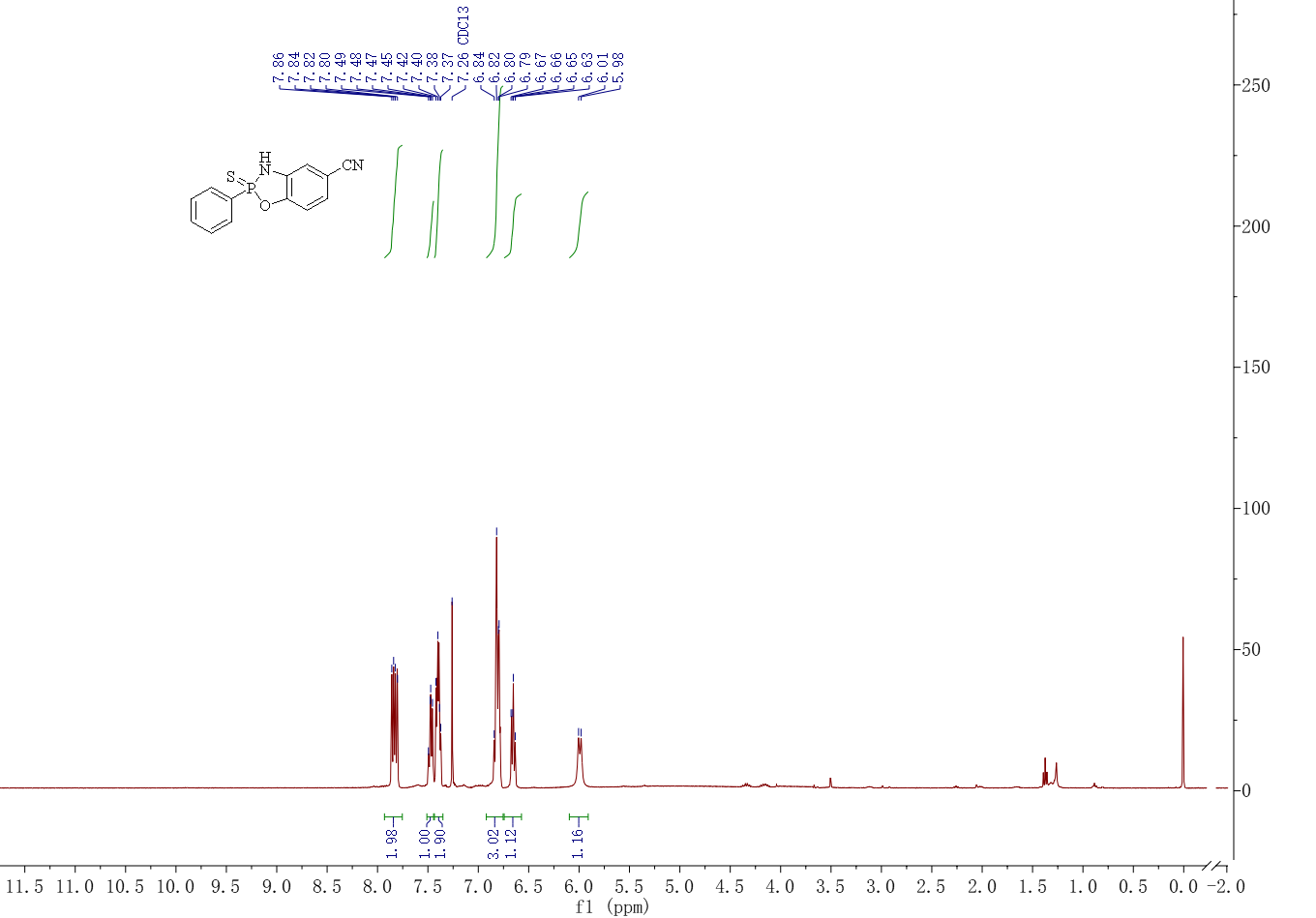


Fig 26. *^1^H NMR of* **3b-3** (400 MHz, CDCl_3_)


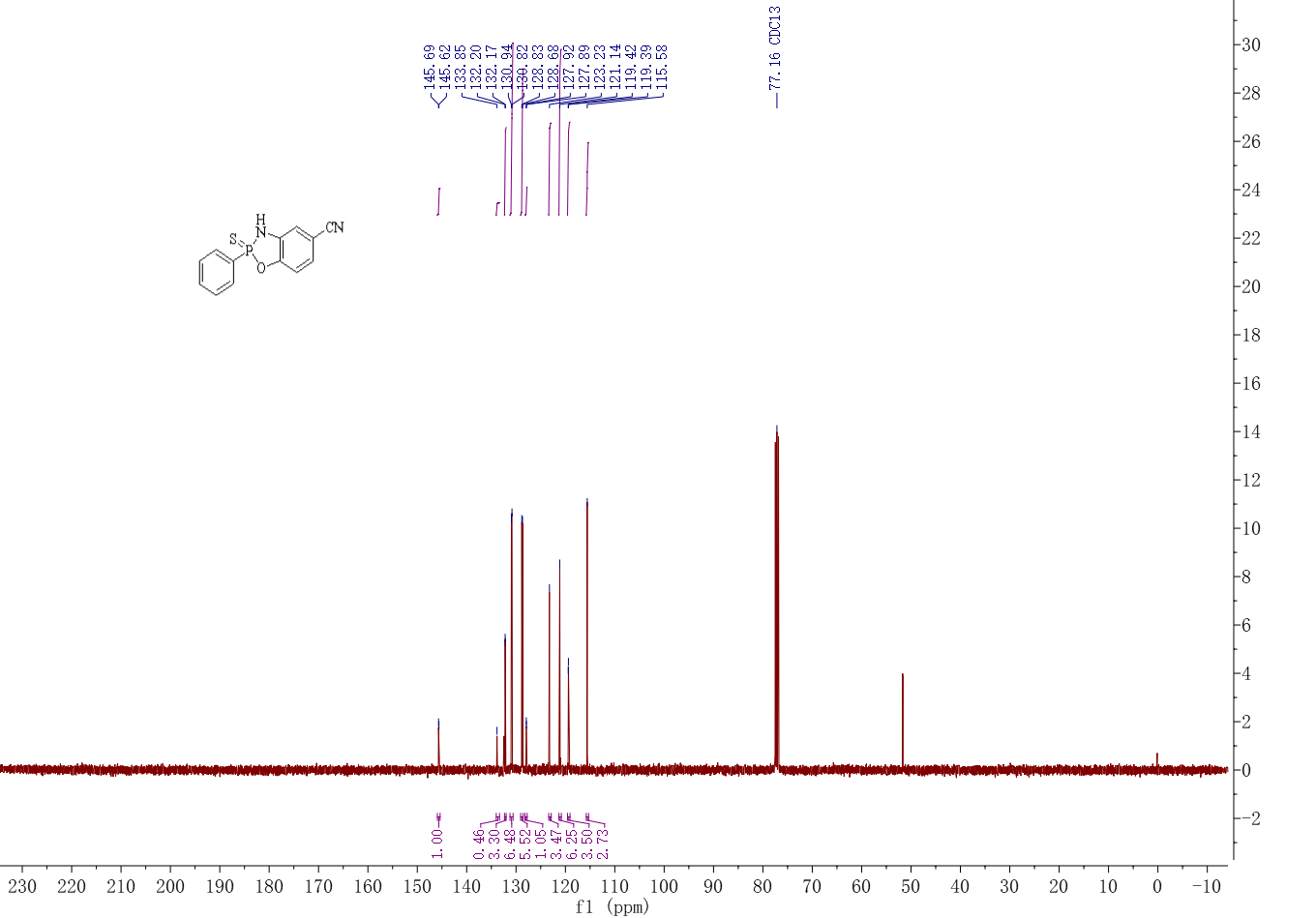


Fig 27. *^13^C NMR of* **3b-3** (101 MHz, CDCl_3_)


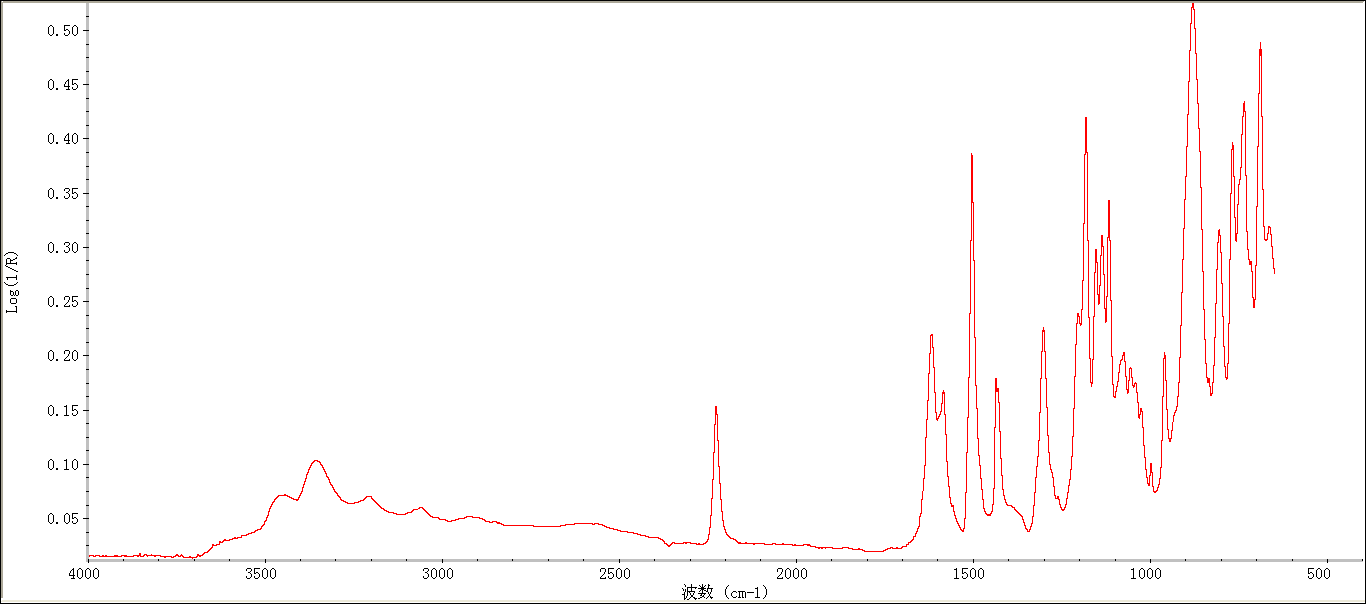


Fig 28. The FTIR spectrum of **3b-3**


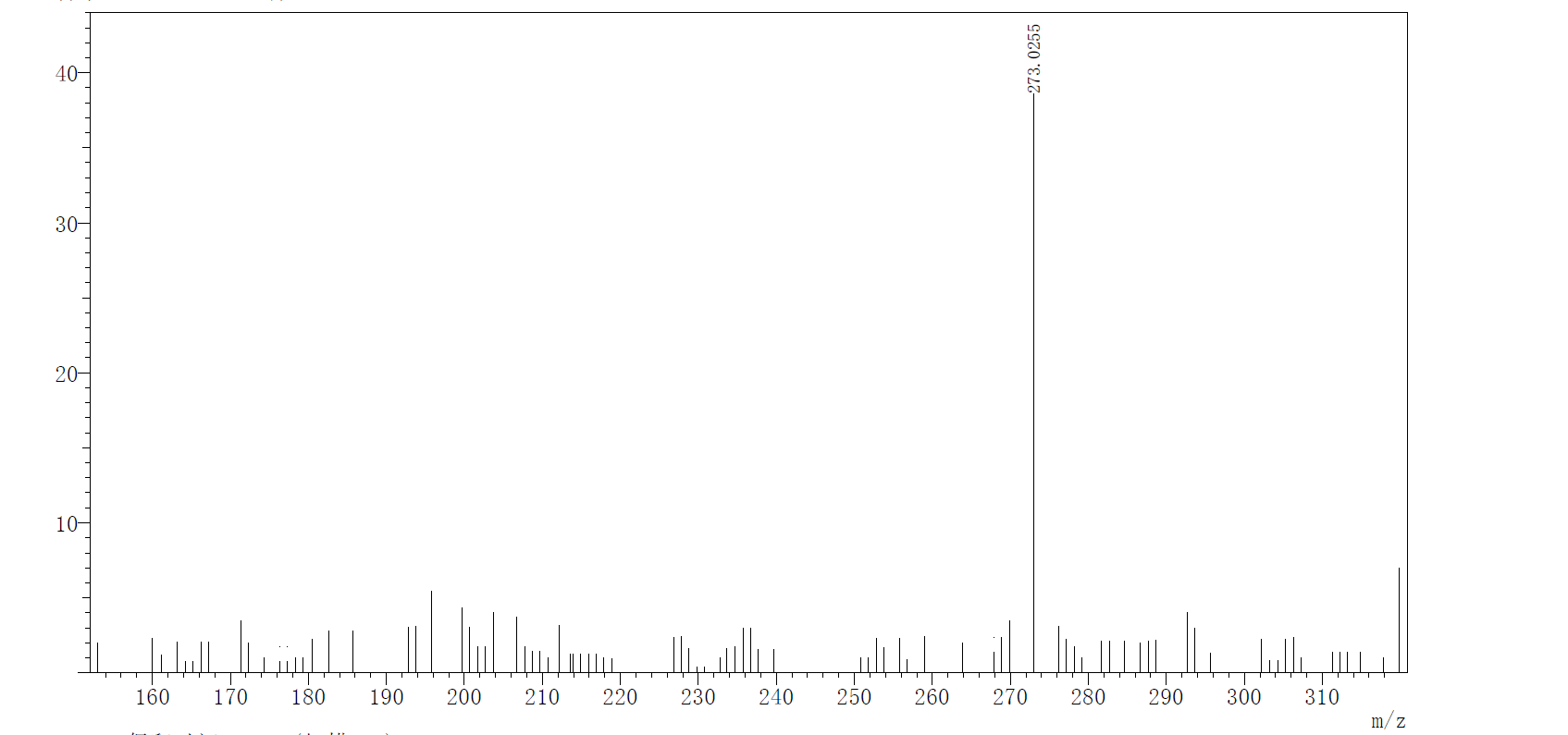


Fig 29. *Mass spectrum of compound* **3b-3**


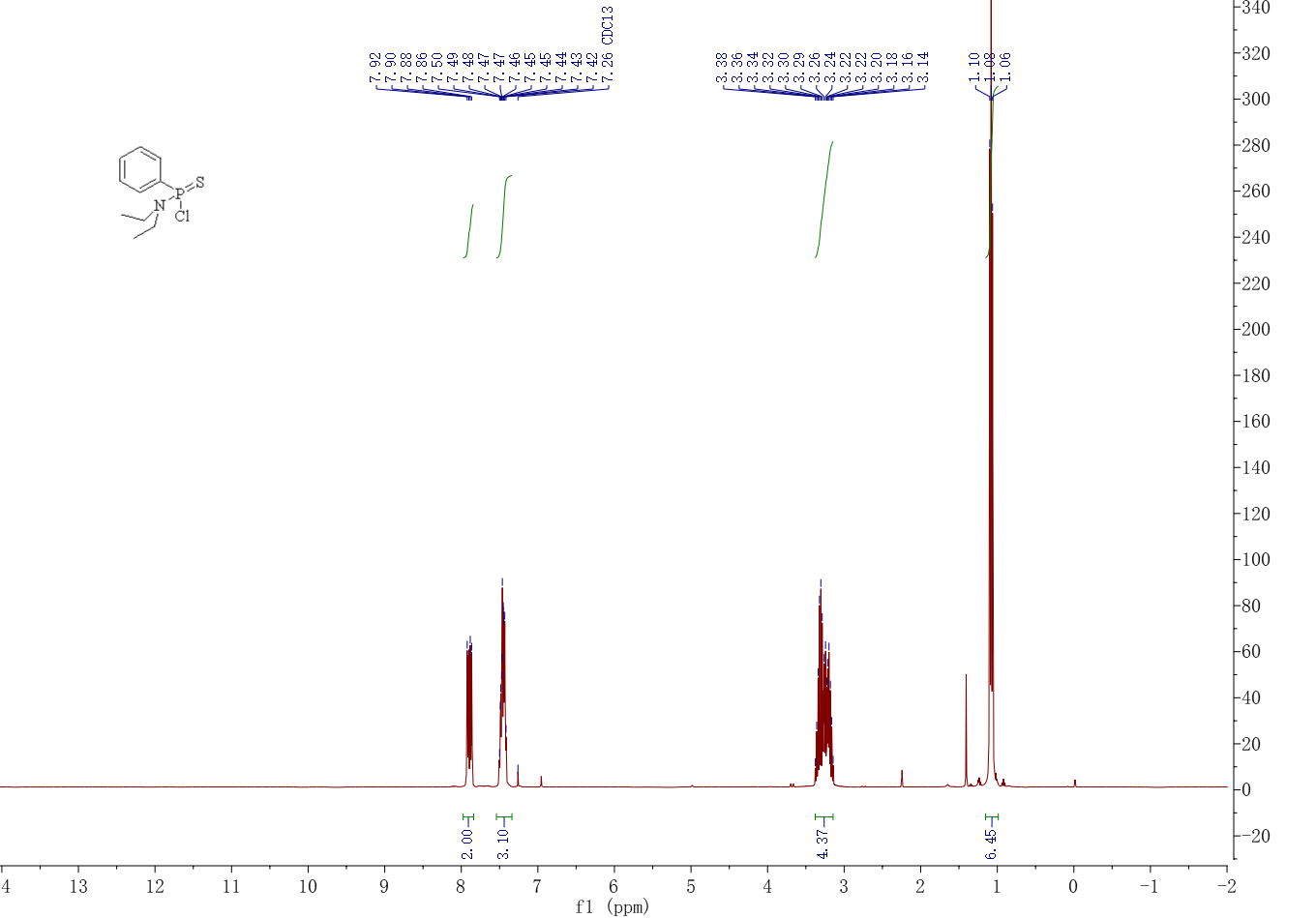


Fig 30. *^1^H NMR of* **3c-1** (400 MHz, CDCl_3_)


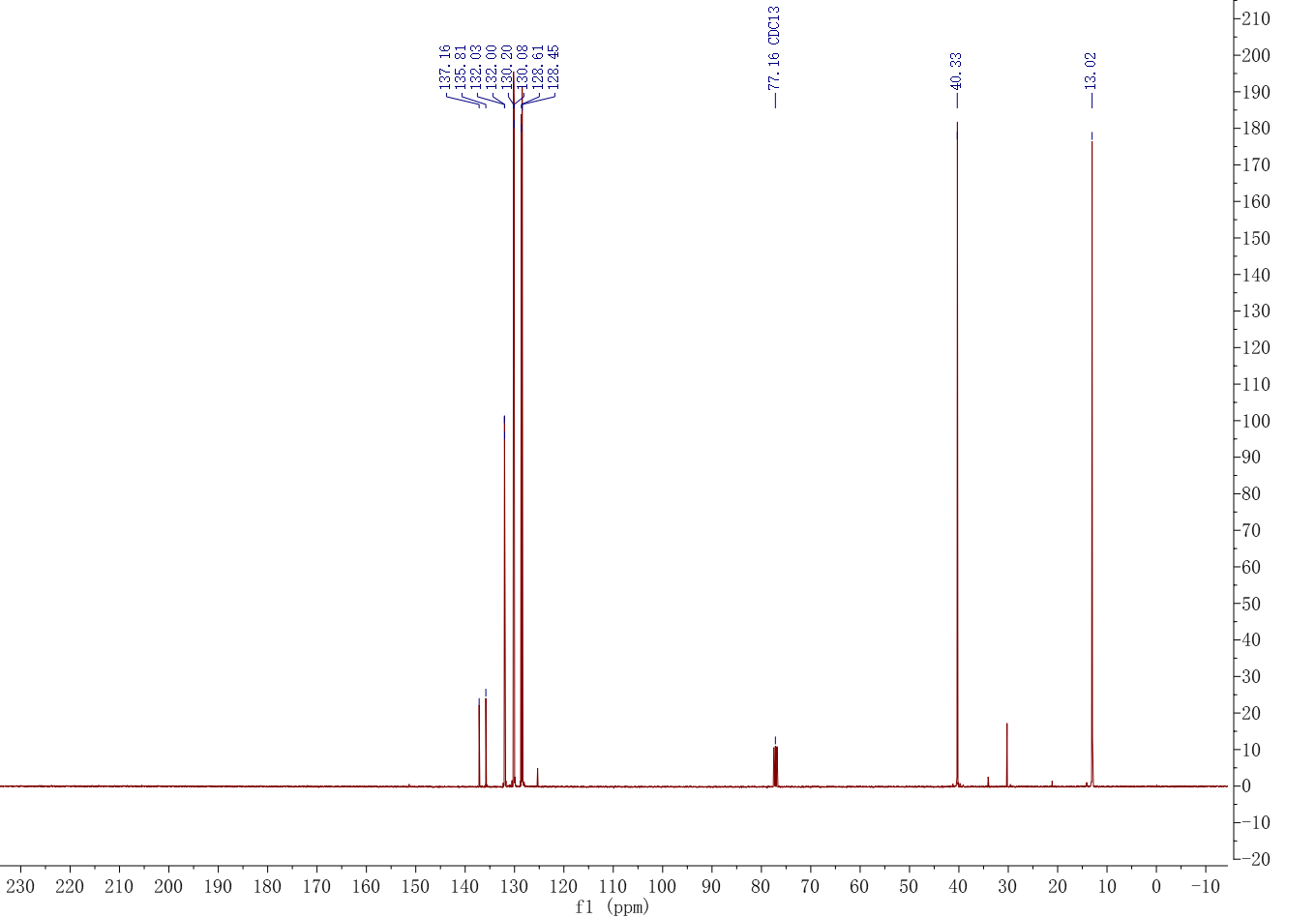


Fig 31. *^13^C NMR of* **3c-1** (101 MHz, CDCl_3_)


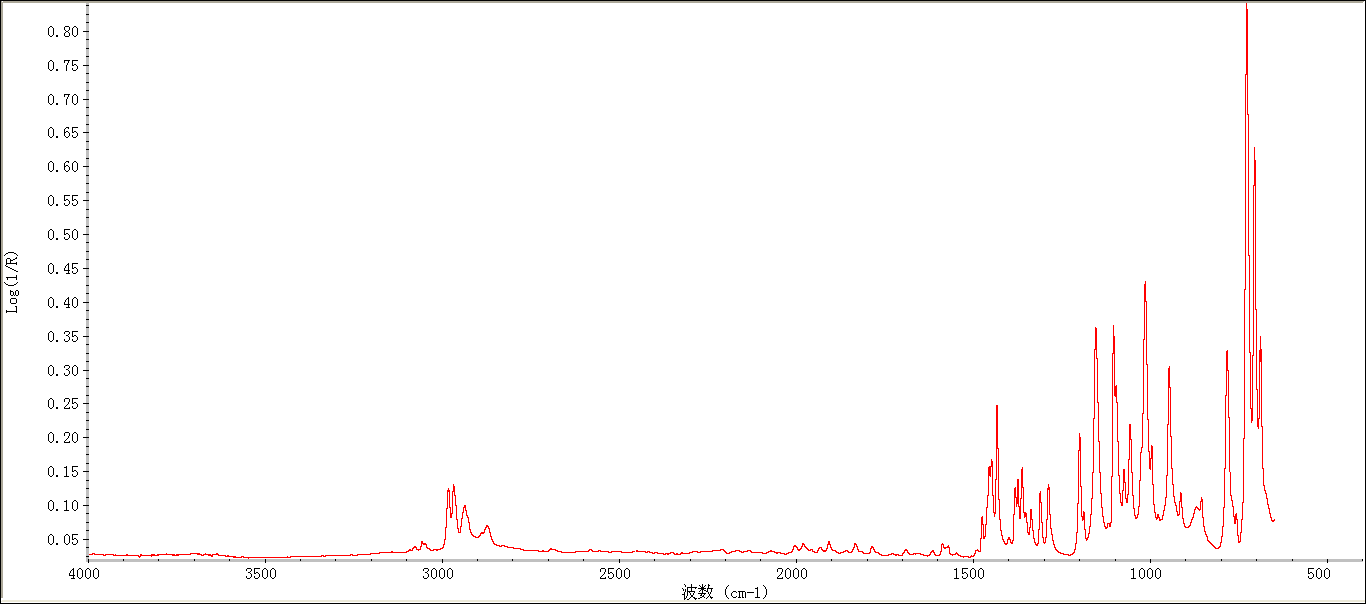


Fig 32. The FTIR spectrum of **3c-1**


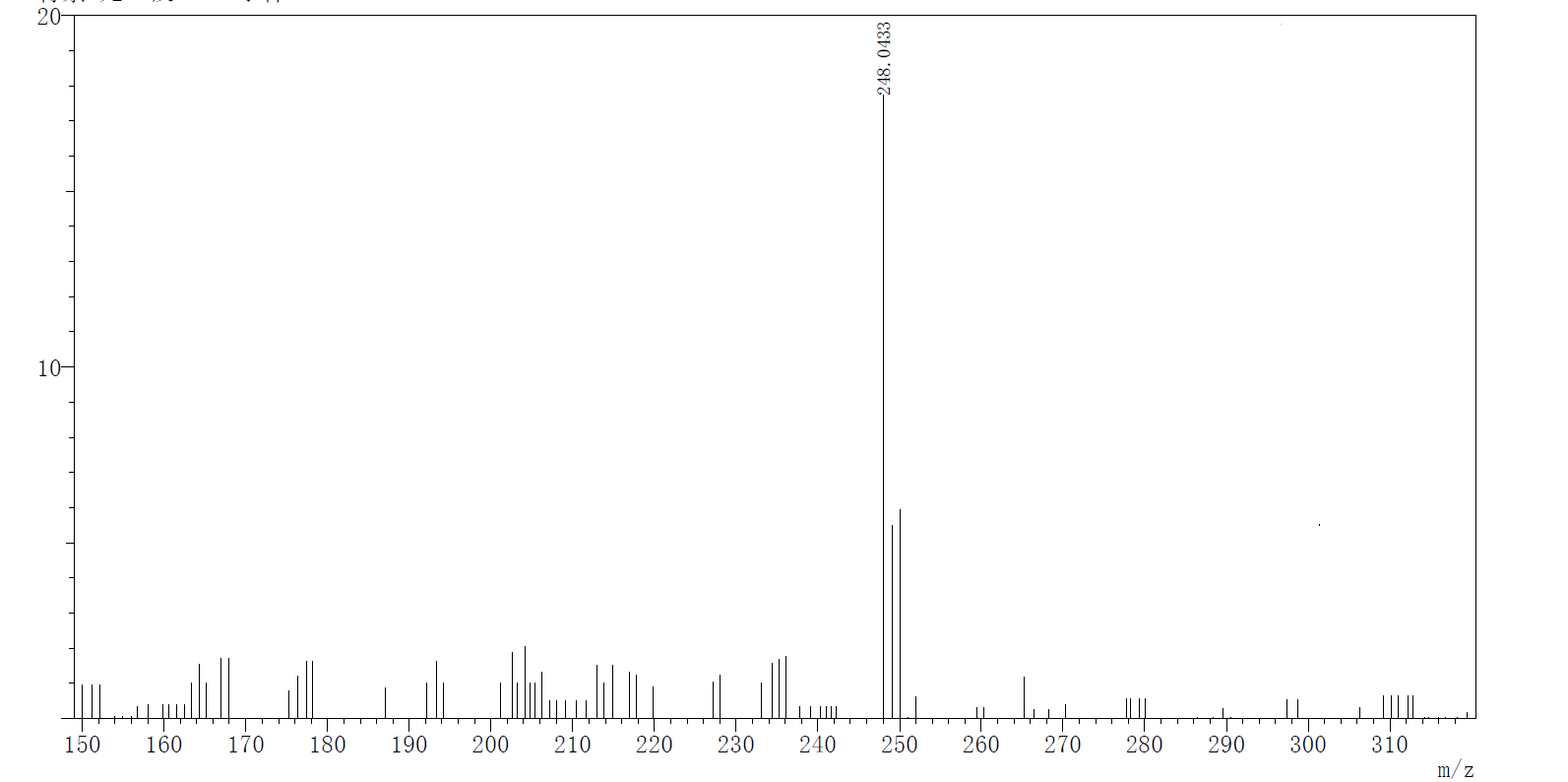


Fig 33. *Mass spectrum of compound* **3c-1**


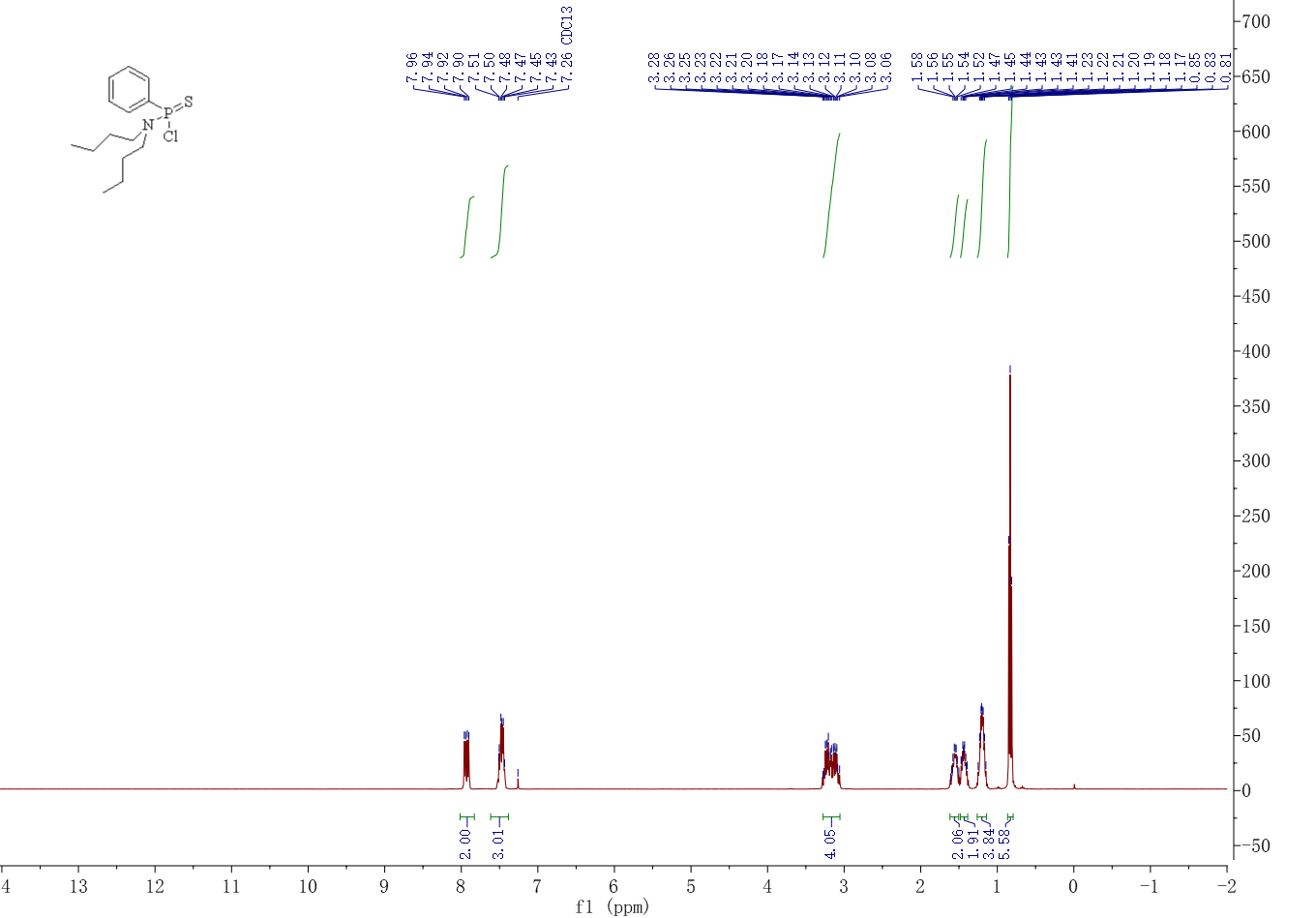


Fig 34. *^1^H NMR of* **3c-2** (400 MHz, CDCl_3_)


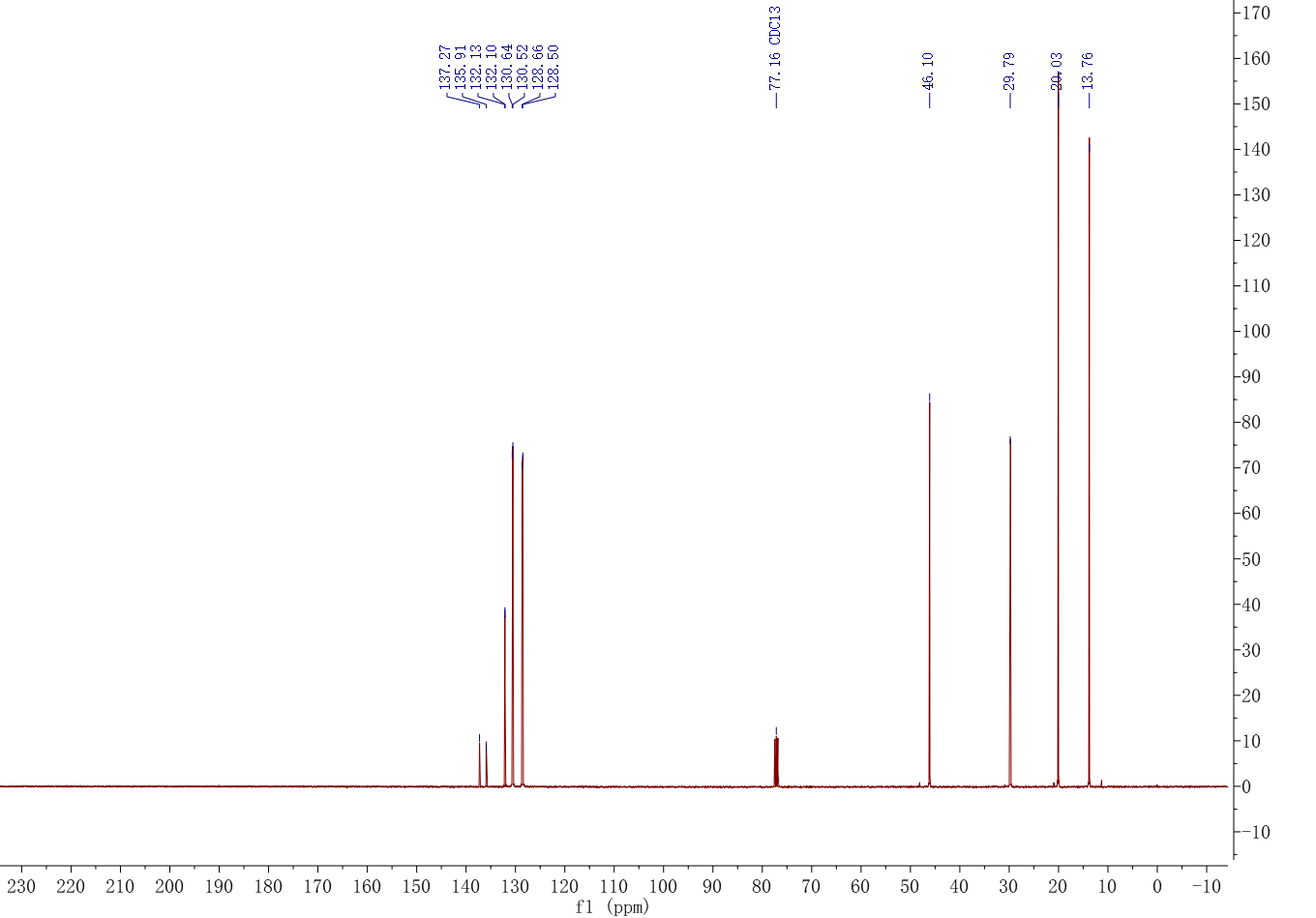


Fig 35. *^13^C NMR of* **3c-2** (101 MHz, CDCl_3_)


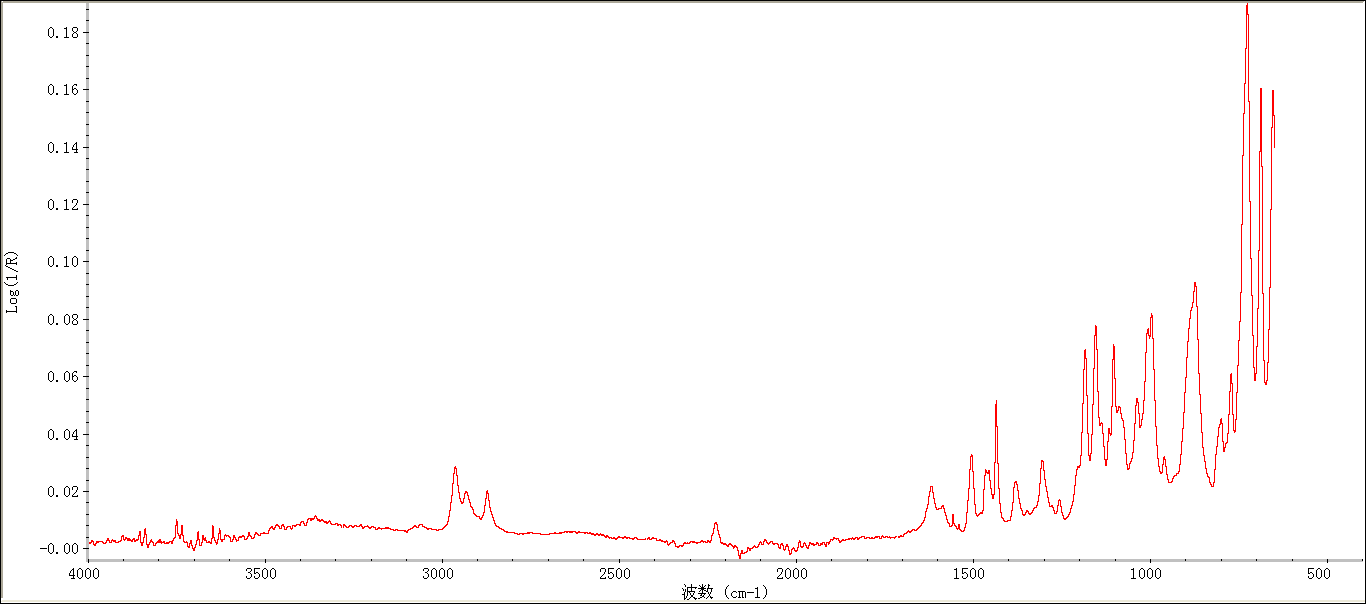


Fig 36. The FTIR spectrum of **3c-2**


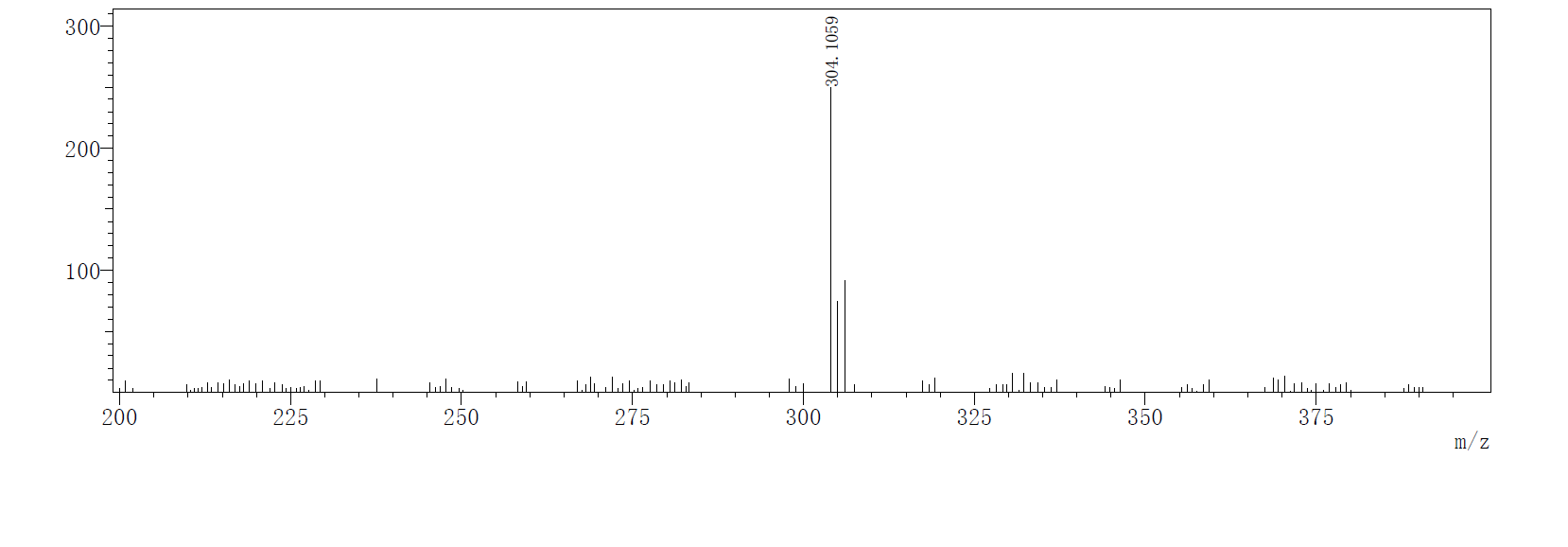


Fig 37. *Mass spectrum of compound* **3c-2**


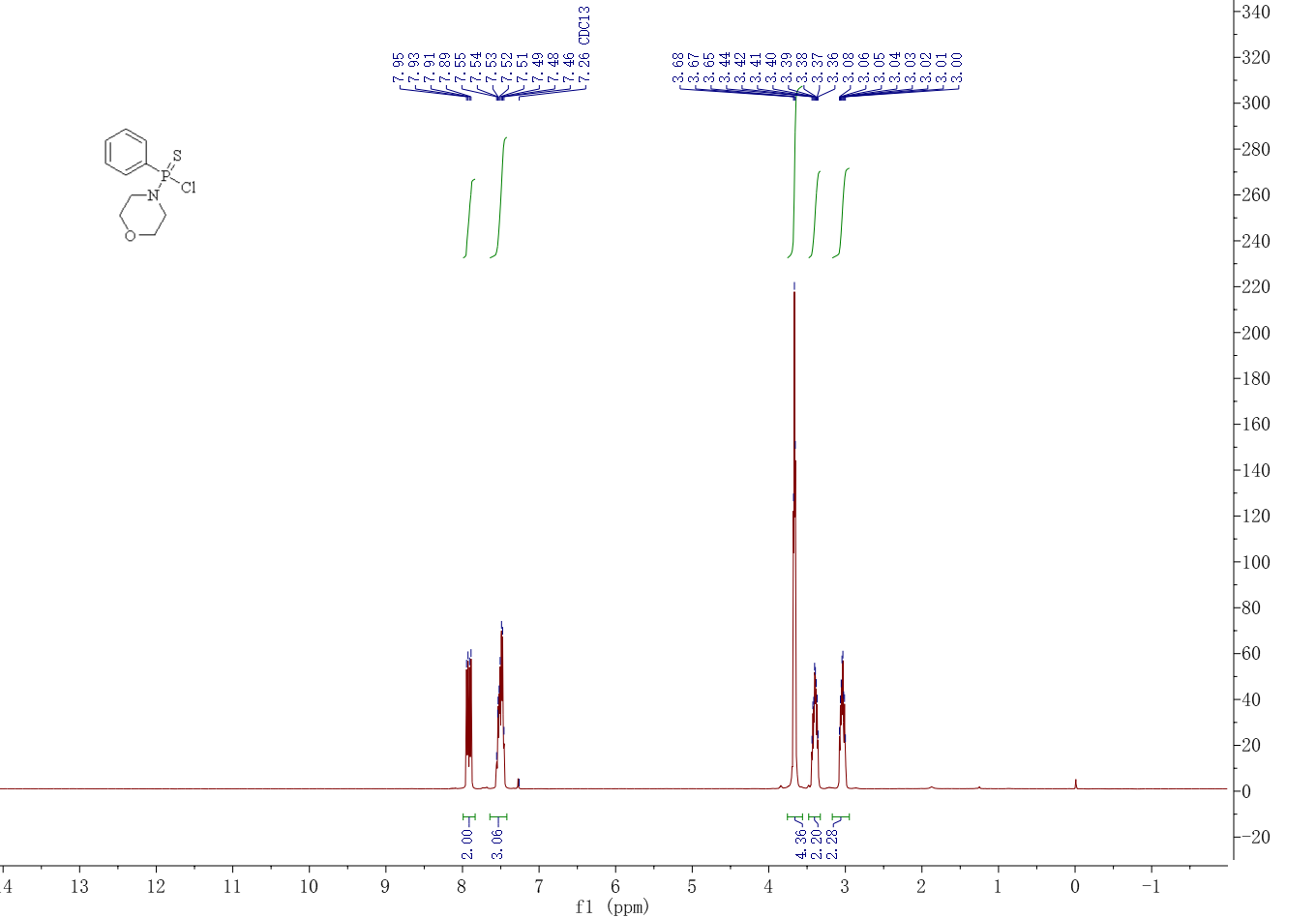


Fig 38. *^1^H NMR of* **3c-3** (400 MHz, CDCl_3_)


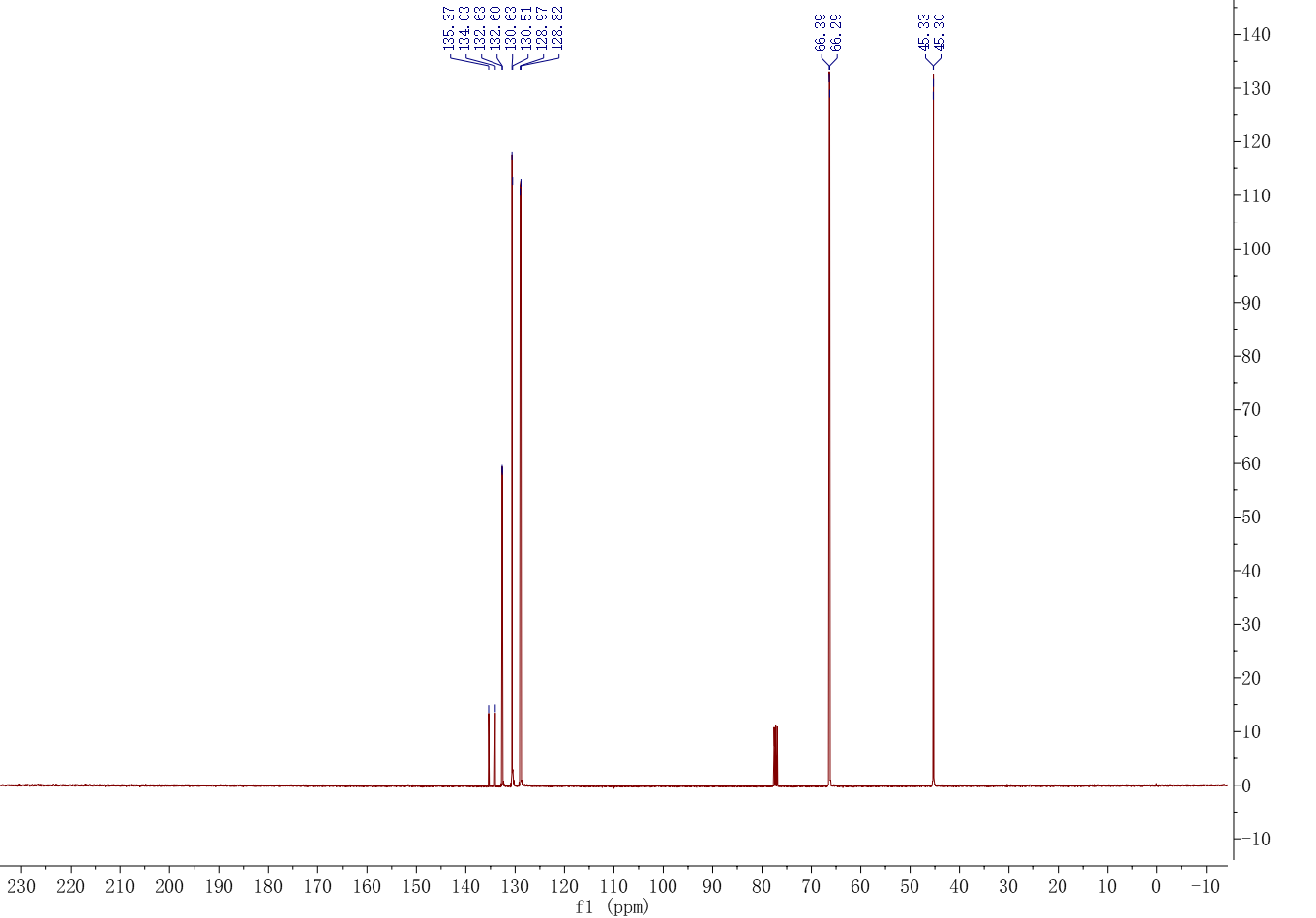


Fig 39. *^13^C NMR of* **3c-3** (101 MHz, CDCl_3_)


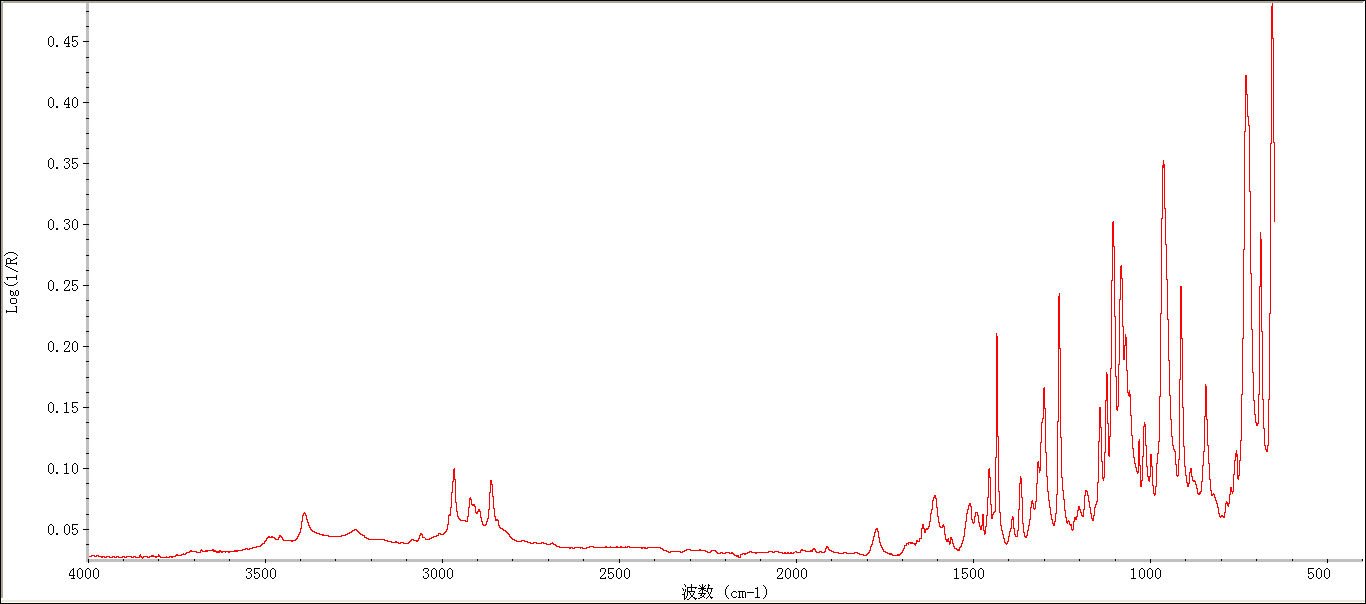


Fig 40. The FTIR spectrum of **3c-3**


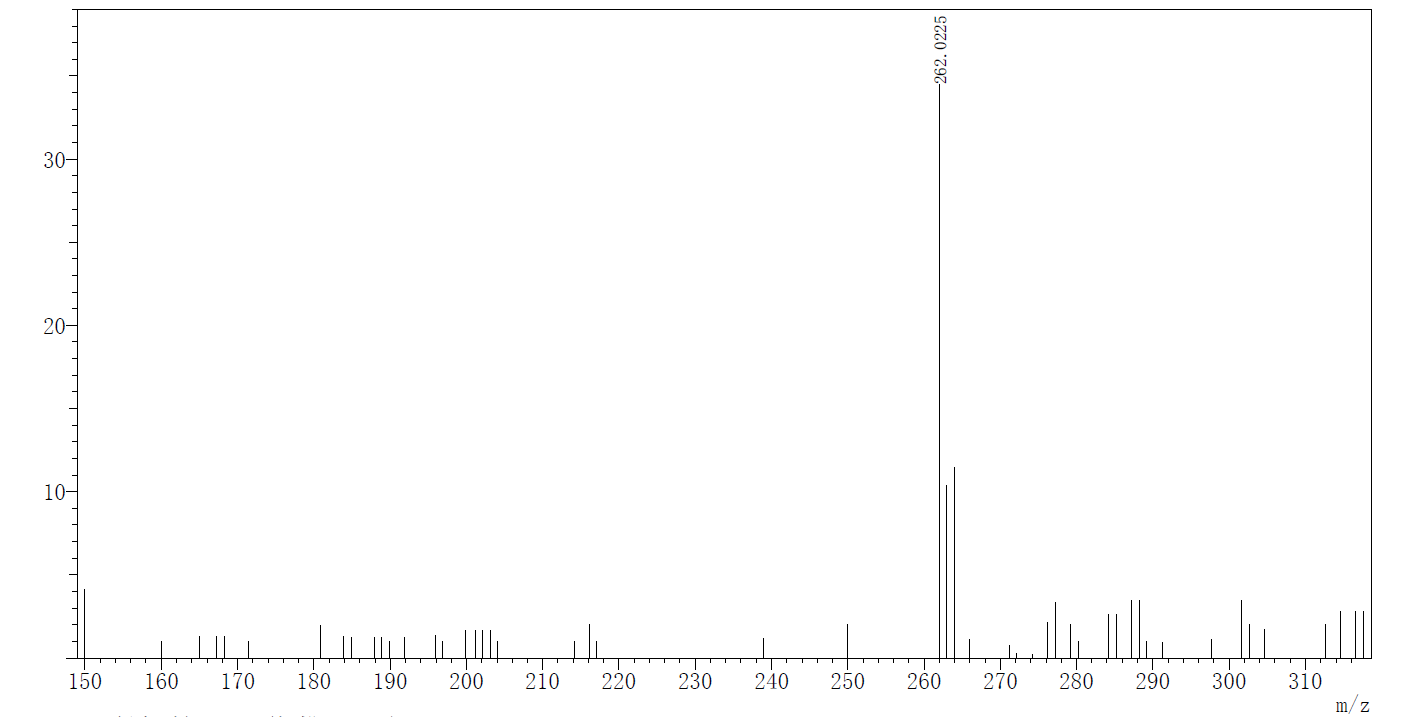


Fig 41. *Mass spectrum of compound* **3c-3**

1. * Correspondent. E-mail: wdh991408@163.com [↑](#footnote-ref-2)
